# Heatstroke predictions by machine learning, weather information, and an all-population registry for 12-hour heatstroke alerts

**DOI:** 10.1038/s41467-021-24823-0

**Published:** 2021-07-28

**Authors:** Soshiro Ogata, Misa Takegami, Taira Ozaki, Takahiro Nakashima, Daisuke Onozuka, Shunsuke Murata, Yuriko Nakaoku, Koyu Suzuki, Akihito Hagihara, Teruo Noguchi, Koji Iihara, Keiichi Kitazume, Tohru Morioka, Shin Yamazaki, Takahiro Yoshida, Yoshiki Yamagata, Kunihiro Nishimura

**Affiliations:** 1 Department of Preventive Medicine and Epidemiology, National Cerebral and Cardiovascular Center, Suita, Osaka Japan; 2 Department of Civil, Environmental and Applied Systems Engineering, Faculty of Environmental and Urban Engineering, Kansai University, Suita, Osaka Japan; 3 Department of Cardiovascular Medicine, National Cerebral and Cardiovascular Center, Suita, Osaka Japan; 4 Director General, National Cerebral and Cardiovascular Center Hospital, Suita, Osaka Japan; 5 Health and Environmental Risk Division, National Institute for Environmental Studies, Tsukuba, Ibaraki Japan; 6 Earth System Division, National Institute for Environmental Studies, Tsukuba, Ibaraki Japan; 7 Department of Urban Engineering, School of Engineering, The University of Tokyo, Tokyo, Japan; 8 Graduate School of System Design and Management, Keio University, Yokohama, Kanagawa Japan

**Keywords:** Machine learning, Environmental health, Public health

## Abstract

This study aims to develop and validate prediction models for the number of all heatstroke cases, and heatstrokes of hospital admission and death cases per city per 12 h, using multiple weather information and a population-based database for heatstroke patients in 16 Japanese cities (corresponding to around a 10,000,000 population size). In the testing dataset, mean absolute percentage error of generalized linear models with wet bulb globe temperature as the only predictor and the optimal models, respectively, are 43.0% and 14.8% for spikes in the number of all heatstroke cases, and 37.7% and 10.6% for spikes in the number of heatstrokes of hospital admission and death cases. The optimal models predict the spikes in the number of heatstrokes well by machine learning methods including non-linear multivariable predictors and/or under-sampling and bagging. Here, we develop prediction models whose predictive performances are high enough to be implemented in public health settings.

## Abstract (web summary)

In the context of climate change, heatstroke is expected to become an increasingly relevant public health concern. Here, the authors develop and validate prediction models for the number of all heatstroke cases in different cities in Japan.

## Introduction

An alert system for heatstroke risk is in urgent need as the mean annual occurrence of extremely hot days in Japan will possibly increase by 1.8 times under a global warming level of 2 °C above pre-industrial levels^1^. Heatstroke, one of the most threatening heat-related illnesses, is characterized by a core temperature of more than 40 °C (104℉) and central nervous system abnormalities^2^, and is strongly associated with weather conditions, especially extremely high temperature and high humidity^3–5^. In fact, serious cases of the heat-related illnesses including heatstroke frequently occurred in 2019 in France, Belgium, and Germany, and in 2018 in Japan, which was caused by extremely hot weather and heat waves^1,6^. Additionally, Japan had many days of extremely high temperature (i.e., daily maximum temperatures exceeding 35 °C) in 2018 (Supplementary Fig. 1)^1^. Accurate predictive values of the number of heatstroke patients must be necessary and can serve as a foundation to optimize medical resources such as assignment of medical staff and ambulances in emergency medicine and public health settings. An alert system based on accurate predictive values can enable us to inform citizens of daily risks of heatstroke and support self-management for high-risk citizens.

Wet bulb globe temperature (WBGT) has been commonly used to assess hot thermal environments and risks of heat-related illnesses including heatstroke^7–9^, though WBGT has several limitations^7^. WBGT cannot precisely stratify the risk of heatstroke incidence in July and August in Japan because there are typically many days on which the maximum WBGT within a day is between 28 °C and 31 °C, and higher than 31 °C, respectively, corresponding to “severe warning (e.g., avoid direct sunlight outdoors)” and “threat of heat disorder (e.g., avoid outdoor activity, and stay in a cool room)”^10^. Additionally, WBGT does not reflect heat strain adequately when the evaporation of sweat is restricted by high humidity or low air movement^7^. Considering these limitations of WBGT, multiple variables related to heat stress should be used to assess heatstroke risks accurately^7^. There are a few studies using multiple weather information to develop prediction models for heat-related illnesses including heatstroke^11,12^. However, the previous models did not consider the severity levels of the heatstroke. Note that it is key to predict the days when the number of heatstrokes spike in public health settings, though this has not been well investigated yet.

Thus, the present study aimed to develop and validate prediction models for the number of all heatstroke cases and heatstrokes of hospital admission (i.e., moderate and severe cases) and death cases per city per 12 h in Japan by using multiple weather information and a population-based database for all heatstroke incidences in 16 Japanese cities corresponding to around a 10,000,000 population size. Here, we report prediction models for the number of heatstroke cases, of which predictabilities were high enough to be implemented in public health settings.

## Results

### Characteristics of the training and testing datasets

We summarized the characteristics of the present training and testing datasets in Table 1. Incidence rates of all heatstrokes (95% confidence interval [CI]) between June and September were 37.5 (36.8–38.2) and 74.4 (72.7–76.1) per 100,000 people in the training and testing datasets, respectively. Those of heatstrokes of hospital admission and death cases were 11.0 (10.6–11.3) and 19.6 (18.8–20.5) in the training and testing datasets, respectively. Median (minimum to maximum) ambient temperatures were 25.52 °C (10.18–33.58) and 26.27 °C (16.01–35.17) in the training and testing datasets, respectively. Based on WBGT, there were 64.2% and 62.7% days for “Severe warning (28–31 °C)” in August of the training and testing datasets; furthermore, there were 24.5% and 30.2% for “Threat of heat disorder (higher than 31 °C) in August of the training and testing datasets. Additionally, mean values of daily maximum temperature were higher in 2018 compared to between 2015 and 2017, which was especially unusual in July (Supplementary Fig. 1). We also showed mean values of temperature, relative humidity, and WBGT in each of the 16 cities in the training and testing datasets (Supplementary Table 1). For the location of the 16 cities, please see Supplementary Fig. 2.

**Table 1:** Characteristics of present datasets in 16 Japanese cities^a^ between June and September from 2015 to 2018.

Dataset	Training dataset	Testing dataset
Year	2015–2017	2018
*Characteristics of heatstroke patients*		
Heatstrokes with all cases		
Total number of heatstrokes, *n*	11349	7513
Incidence rate (95% CI) between June and September per 100,000 persons	37.5 (36.8–38.2)	74.4 (72.7–76.1)
Heatstrokes of hospital admission (i.e., moderate and severe cases) and death cases		
Total number of heatstrokes, *n*	3318	1981
Incidence rate (95% CI) between June and September per 100,000 persons	11.0 (10.6–11.3)	19.6 (18.8–20.5)
*Weather information, median (minimum, maximum)*^*b*^ *per 12* *h*		
Temperature, °C	25.52 (10.18–33.58)	26.27 (16.01–35.17)
Heat index, °C	25.86 (10.18–38.76)	26.91 (16.01–40.93)
WBGT, °C	23.72 (9.22–30.56)	24.13 (13.78–31.31)
Relative humidity, %	73.2 (35.15–99.79)	70.96 (38.51–99.75)
Precipitation previous 12 h, mm	0.01 (0–212.78)	0 (0–391.56)
Downward solar radiation, kW/m^2^	0.02 (0–0.71)	0.02 (0–0.69)
Wind speed, m/s	2.5 (0.44–11.69)	2.62 (0.33–14.48)
*Days warned by WBGT guides to prevent heatstrokes in daily life, %*		
July		
Warning (25–28 °C)	14.8	10.1
Severe warning (28–31 °C)	76.0	42.7
Threat of heat disorder (higher than 31 °C)	6.0	43.1
August		
Warning (25–28 °C)	11.2	6.9
Severe warning (28–31 °C)	64.2	62.7
Threat of heat disorder (higher than 31 °C)	24.5	30.2

*CI* confidence interval, *WBGT* wet bulb globe temperature.

^a^ The 16 cities were Osaka, Toyonaka, Mino, Ikeda, Suita, Sakai, Kobe, Ashiya, Nishinomiya, Amagasaki, Akashi, Himeji, Kyoto, Uji, Muko, and Nagaokakyo that located in the Kinki region in Japan.

^b^ Median (minimum, maximum) values were based on mean values per city per 12 h (6:00 am–5:59 pm, and 6:00 pm–5:59 am).

### Prediction models for all heatstroke cases

**Fig. 1:**
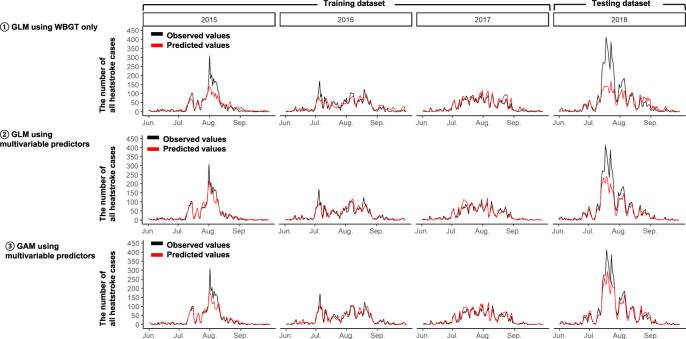
Comparison between observed and predicted numbers of all heatstroke cases from June to September in 2015, 2016, and 2017 (i.e., training dataset), and 2018 (i.e., testing dataset) by GLMs and GAM. The black lines indicate the observed total number of heatstroke per day in the 16 Japanese cities, and the red lines indicate the predicted total number of heatstroke per day in the 16 Japanese cities obtained from the following prediction models: (1) GLM using WBGT as the only predictor, (2) GLM using multivariable predictors, and (3) GAM using multivariable predictors. GLM generalized linear model, GAM generalized additive model, WBGT wet bulb globe temperature.

We developed prediction models for the number of all heatstrokes by the generalized linear model (GLM) using WBGT only as a classic statistical model, and by machine learning models including GLM, generalized additive model (GAM), random forest, and extreme gradient boosting decision tree (XGBoost) using multivariable predictors in the training dataset (Table 2). Among the developed models, the GAM was the best model based on the least root-mean squared error (RMSE). RMSEs of the GLM using WBGT only, the GLM using multivariable predictors as linear terms, and the GAM using multivariable predictors as non-linear terms were, respectively, 3.73, 2.92, and 2.47 in the testing dataset (Table 2). These results are also graphically shown in Fig. 1. The observed number of all heatstroke cases was strongly correlated with the predicted number by those prediction models (Supplementary Fig. 3). RMSE of the best GAM was also lower than RMSEs of the random forest (3.51 in the testing) and the XGBoost (3.28 in the testing). In the best GAM, all predictors were selected as the optimal predictors’ set by recursive feature elimination (RFE).

**Table 2:** Prediction performances of prediction models for the number of all heatstrokes among 6 models^a^.

	GLM using WBGT only	GLM	GAM	RF	XGBoost	Consolidation of 16 GAMs specific to each city^b^
	The number of all heatstrokes					
*Overall predictive accuracies per city per 12* *h*						
RMSE in training	1.73	1.41	1.37	0.73	1.09	1.27
RMSE in testing	3.73	2.92	2.47	3.51	3.28	5.25
*Predictive accuracies on days when the number of heatstrokes spiked*^*c*^						
MAPE per 1-day (%) in training	22.6	18.4	18.0	8.3	11.9	16.8
MAPE per 1-day (%) in testing	43.0	27.1	19.7	32.0	28.5	19.0
Total absolute percentage error (%) in training	18.8	7.3	8.3	5.5	5.9	7.5
Total absolute percentage error (%) in testing	48.8	30.5	21.9	37.2	31.9	19.7

*GLM* generalized linear model, *GAM* generalized additive model, *RF* random forest, *XGBoost* extreme gradient boosting decision tree, *WBGT* wet bulb globe temperature, *RMSE* root-mean-square error, *MAPE* mean absolute percentage error.

^a^Smaller RMSE, MAPE, and total absolute percentage error show better predictabilities.

^b^Prediction models specific to each of 16 cities were developed for city-specific prediction.

^c^MAPE and total absolute percentage error were calculated after observed and predicted values were summed up per day (for MAPE) per the entire period (for total absolute percentage error) on days when the number of all heatstrokes was 80th percentile (corresponding to 53.6 in 2015, 57.8 in 2016, 60.6 in 2017, and 89.8 in 2018) and over in each year. MAPE is a mean value of absolute errors divided by observed values.

Based on the results, we also developed 16 GAMs for city-specific prediction as city-specific prediction models. RMSE of the total 16 GAMs for city-specific prediction (i.e., summarized across all cities), were higher compared with the GAM not specific to each city (5.25 versus 2.47 in the testing, Table 2). Additionally, RMSEs of the 16 GAMs for city-specific prediction were similar in the training and testing data of almost all cities, but higher in the testing data of Kobe and Sakai, compared to the GAM not specific to each city (Supplementary Table 2). Thus, we considered that the GAM not specific to each city was better than the 16 GAMs specific to each city. Additionally, the GAM not specific to each city had no serious heterogeneity.

**Fig. 2:**
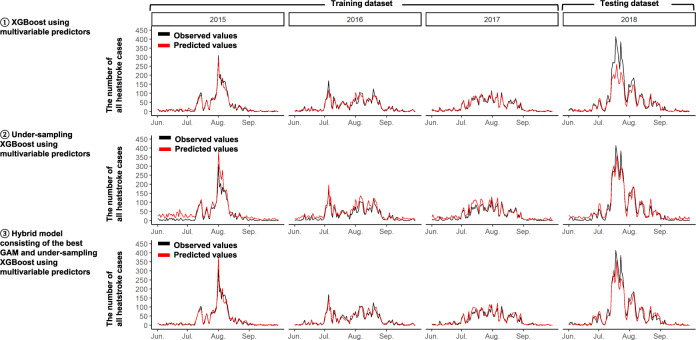
Comparison between observed and predicted numbers of all heatstroke cases from June to September in 2015, 2016, and 2017 (i.e., training dataset), and 2018 (i.e., testing dataset) by XGBoost models and hybrid model. The black lines indicate the observed total number of heatstroke per day in the 16 Japanese cities, and the red lines indicate the predicted total number of heatstroke per day in the 16 Japanese cities obtained from the following prediction models: (1) XGBoost using multivariable predictors, (2) Under-sampling XGBoost using multivariable predictors, and (3) Hybrid model consisting of the best GAM and under-sampling XGBoost using multivariable predictors. *GAM* generalized additive model, *XGBoost* extreme gradient boosting decision tree.

However, the best GAM had low accuracy in predicting spikes in the number of all heatstroke cases (Fig. 1). In fact, its mean absolute percentage error (MAPE) and total absolute percentage error on days when the number of all heatstroke cases spiked were, respectively, 18.0% and 8.3% in the training, and 19.7% and 21.9% in the testing datasets (Table 2). Thus, we applied the under-sampling and bagging techniques to the best GAM. Compared with the best GAM and the XGBoost model, the best under-sampling XGBoost model had better accuracy in predicting spikes in the number of all heatstroke cases based on lower MAPE (13.38% in the testing) and lower total absolute percentage error (6.94% in the testing) in Table 3 and Fig. 2. However, the best under-sampling XGBoost model had bad prediction accuracy at normal times other than where spikes occur (Fig. 2), which we anticipated a priori. Thus, we made the hybrid model consisting of the best GAM and the best under-sampling XGBoost model, of which RMSE, MAPE, and total absolute percentage error were, respectively, 2.97, 14.8%, and 14.2% in the testing dataset (Table 3). Additionally, its prediction accuracy on days when the number of all heatstrokes cases spiked was also good (Fig. 2). The observed number of all heatstroke cases was strongly correlated with the predicted number by those prediction models (Supplementary Fig. 4). Additionally, city-specific RMSEs of the hybrid model are shown in Supplementary Table 2, similar to RMSEs of the 16 GAMs specific to each city. Thus, the hybrid model had no serious heterogeneity.

**Table 3:** Prediction performances of prediction models related to under-sampling and bagging techniques for the number of all heatstrokes^a^.

	GAM ^b^	XGBoost^b^	Under-sampling XGBoost model	Hybrid model consisting of GAM and under-sampling XGBoost model
	The number of all heatstrokes			
*Overall predictive accuracies per city per 12* *h*				
RMSE in training	1.37	1.09	1.48	1.28
RMSE in testing	2.47	3.28	3.48	2.97
*Predictive accuracies on days when the number of heatstrokes spiked*^*c*^				
MAPE per 1-day (%) in training	18.0	11.9	23.81	16.3
MAPE per 1-day (%) in testing	19.7	28.5	13.38	14.8
Total absolute percentage error (%) in training	8.3	5.9	20.79	1.2
Total absolute percentage error (%) in testing	21.9	31.9	6.94	14.2

*GAM* generalized additive model, *XGBoost* extreme gradient boosting decision tree, *RMSE* root-mean-square error, *MAPE* mean absolute percentage error.

^a^ Smaller RMSE, MAPE, and total absolute percentage error show better predictabilities.

^b^ These GAM and XGBoost models were the same as those in Table 2.

^c^ MAPE and total absolute percentage error were calculated after observed and predicted values were summed up per day (for MAPE) per the entire period (for total absolute percentage error) on days when the number of all heatstrokes was 80th percentile (corresponding to 53.6 in 2015, 57.8 in 2016, 60.6 in 2017, and 89.8 in 2018) and over in each year. MAPE is a mean value of absolute errors divided by observed values.

**Fig. 3:**
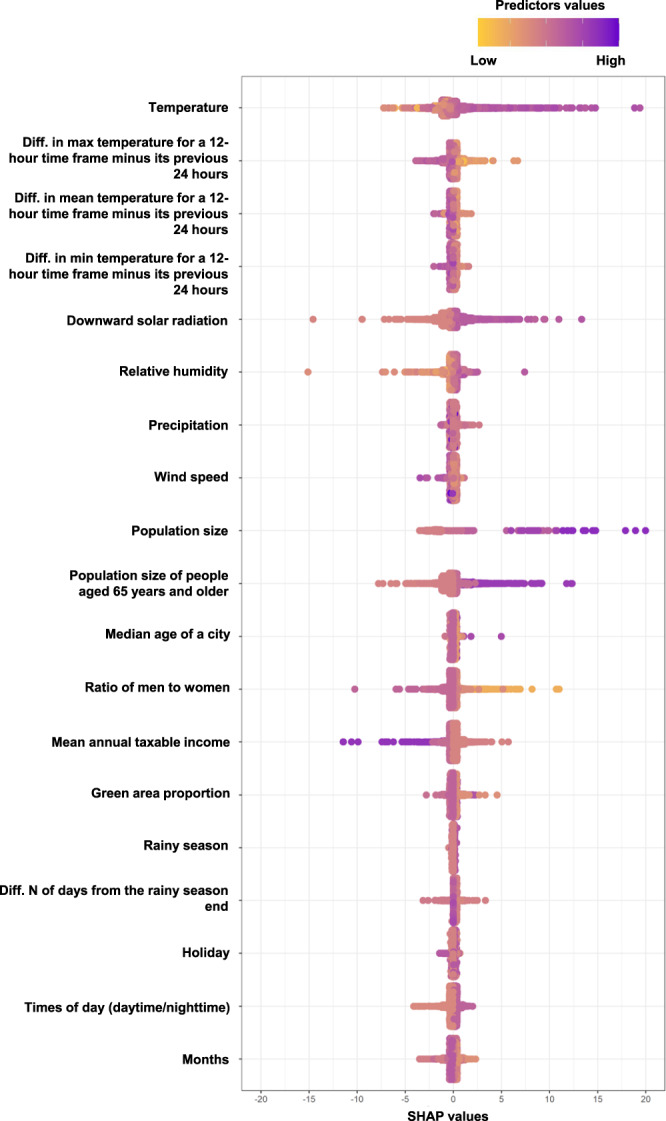
Importance of predictors for the number of all heatstroke cases based on SHAP values in the best model (i.e., the hybrid model). Plots show importance of the predictors in the best prediction models (i.e., the hybrid model consisting of the best GAM and the best under-sampling XGBoost model) by SHAP values. The yellow to purple dots represent low to high values of each predictor. The x-axis shows the SHAP value, the contribution of each predictor to the predicted number of heatstrokes of which positive values tend to predict a higher number of heatstroke cases and negative values tend to predict a lower number of heatstroke cases. When standardized values of wind speed and precipitation were over 5, the values were treated as 5. SHAP values over 20 or under −20 were eliminated, but there were few such values (only 3 cases). SHAP SHapley Additive exPlanations, Diff. N difference number, XGBoost extreme gradient boosting decision tree.

SHapley Additive exPlanations (SHAP) values of the best hybrid model are summarized in Fig. 3 to show importance of its predictors. Predictors that worked for predicting a higher number of all heatstroke cases were high temperature, small difference in maximum temperature for a 12-h time frame minus its previous 24 h (i.e., consecutive hot days), high downward solar radiation, large population size, and large population size of people aged 65 years and older. Additionally, predictors that worked for predicting a lower number of heatstrokes of hospital admission and death cases were low relative humidity, high ratio of men to women, and high mean annual taxable income. We also showed heatmaps for the numbers of all heatstrokes observed and predicted by the best hybrid model in Supplementary Fig. 5, which was summed up across the entire period in the testing dataset.

### Prediction models for heatstrokes of hospital admission and death cases

**Fig. 4:**
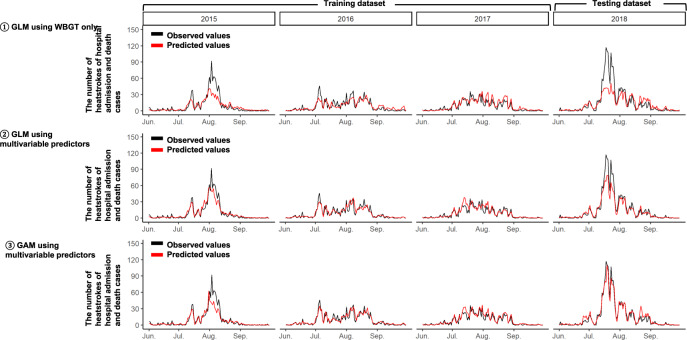
Comparison between observed and predicted numbers of heatstrokes of hospital admission and death cases from June to September in 2015, 2016, and 2017 (i.e., training dataset), and 2018 (i.e., testing dataset) by GLMs and GAM. The black lines indicate the observed total number of heatstroke per day in the 16 Japanese cities, and the red lines indicate the predicted total number of heatstroke per day in the 16 Japanese cities obtained from the following prediction models: (1) GLM using WBGT as the only predictor, (2) GLM using multivariable predictors, and (3) GAM using multivariable predictors. GLM generalized linear model, GAM generalized additive model, WBGT wet bulb globe temperature.

We developed prediction models for the number of heatstrokes of hospital admission and death cases (Table 4). Among the developed models, the GAM was the best model based on the least RMSE. RMSEs of GLM using WBGT only, GLM, and GAM using multivariable predictors were, respectively, 1.14, 0.92, and 0.83 in the testing dataset (Table 4). These results are also graphically shown (Fig. 4). The observed number of heatstrokes of hospital admission and death cases was strongly correlated with the predicted number by those prediction models (Supplementary Fig. 6). RMSE of the best GAM was also lower than RMSEs of the random forest (1.09 in the testing) and the XGBoost model (1.08 in the testing) in Table 4. In the best GAM, all predictors were selected as the optimal set of predictors by RFE.

**Table 4:** Predictabilities of the prediction models for the number of heatstrokes of hospital admission and death cases among 6 models^a^.

	GLM using WBGT only	GLM	GAM	RF	XGBoost	Consolidation of 16 GAMs specific to each city^b^
	The number of heatstrokes of hospital admission and death cases					
*Overall predictive accuracies per city per 12* *h*						
RMSE in training	0.68	0.62	0.62	0.3	0.44	0.61
RMSE in testing	1.14	0.92	0.83	1.09	1.08	1.42
*Predictive accuracies on days when the number of heatstrokes spiked*^*c*^						
MAPE per 1-day (%)^c^ in training	28.3	23.5	23.3	9.4	13.2	23.4
MAPE per 1-day (%)^c^ in testing	37.7	23.7	10.6	21.2	24.9	10.4
Total absolute percentage error (%) in training	21.8	11.5	11.7	5.0	7.2	11.8
Total absolute percentage error (%) in testing	42.9	25.8	7.5	26.9	29.7	2.7

*GLM* generalized linear model, *GAM* generalized additive model, *RF* random forest, *XGBoost* extreme gradient boosting decision tree, *WBGT* wet bulb globe temperature, *RMSE* root-mean-square error, *MAPE* mean absolute percentage error.

^a^ Smaller RMSE, MAPE, and total absolute percentage error show better predictabilities.

^b^ Prediction models specific to each of the 16 cities were developed for city-specific prediction.

^c^ MAPE and total absolute percentage error were calculated after observed and predicted values were summed up per day (for MAPE) per the entire period (for total absolute percentage error) on days when the number of heatstrokes of hospital admission (i.e., moderate and severe cases) and death cases was 80th percentile (corresponding to 15.6 in 2015, 16 in 2016, 17 in 2017, and 23 in 2018) and over in each year. MAPE is a mean value of absolute errors divided by observed values.

Additionally, the best GAM had relatively high accuracy in predicting spikes in the number of heatstrokes of hospital admission and death cases (Fig. 4). In fact, its MAPE and total absolute percentage error on days when the number of heatstrokes of hospital admission and death cases spiked were, respectively, 23.3% and 11.7% in the training, and 10.6% and 7.5% in the testing datasets (Table 4). Thus, we did not need to apply the under-sampling and bagging techniques.

We also developed 16 GAMs for city-specific prediction. RMSE of the total 16 GAMs for city-specific prediction, were higher compared with the GAM not specific to each city (1.42 versus 0.83 in the testing, Table 4). Additionally, RMSEs of the 16 GAMs for city-specific prediction were similar in the training and testing data of the almost all cities, but higher in the testing data of Sakai, compared to the GAM not specific to each city (Supplementary Table 3). Thus, we considered that the GAM not specific to each city was better than the 16 GAMs specific to each city. Additionally, the GAM not specific to each city had no serious heterogeneity.

**Fig. 5:**
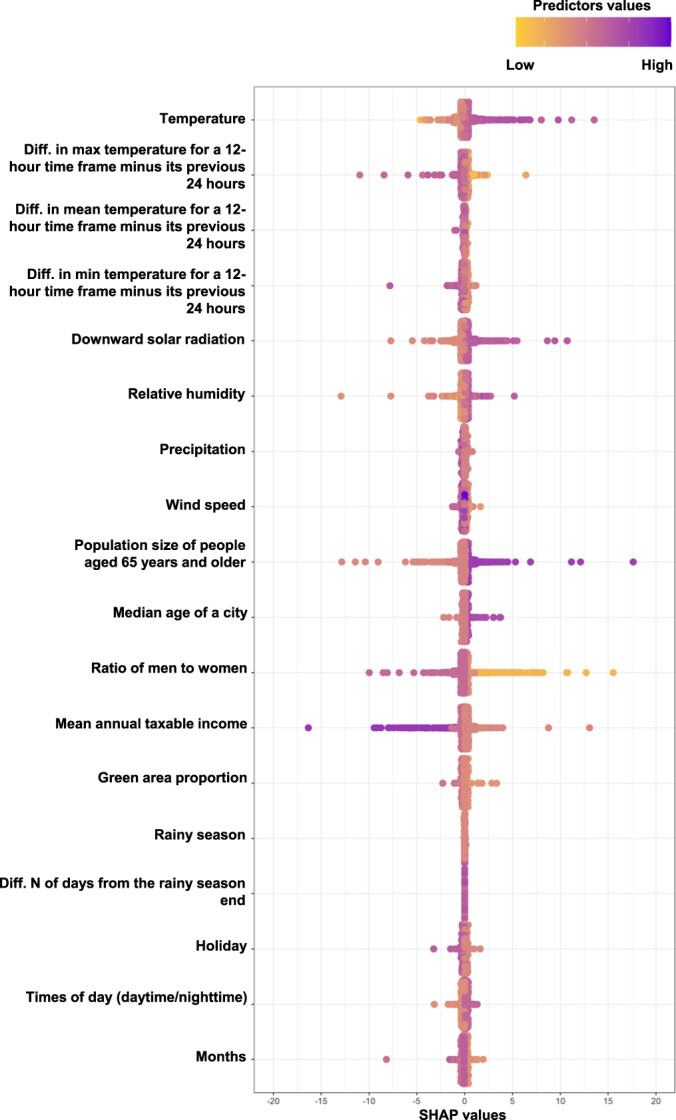
Importance of predictors for the number of heatstrokes of hospital admission and death cases based on SHAP values in the best GAM. Plots show importance of the predictors in the best prediction models (i.e., the GAM using multivariable predictors) by SHAP values. The yellow to purple dots represent low to high values of each predictor. The x-axis shows the SHAP value, the contribution of each predictor to the predicted number of heatstrokes of which positive values tend to predict a higher number of heatstroke cases and negative values tend to predict a lower number of heatstroke cases. When standardized values of wind speed and precipitation were over 5, the values were treated as 5. SHAP values over 20 or under −20 were eliminated, but there were few such values (only 1 case). SHAP SHapley Additive exPlanations, Diff. N difference number, GAM generalized additive model.

SHAP values of the best GAM are summarized in Fig. 5 to show importance of its predictors. Predictors that worked for predicting a higher number of heatstrokes of hospital admission and death cases were high temperature, small difference in maximum temperature for a 12-h time frame minus its previous 24 h (i.e., consecutive hot days), high downward solar radiation, and large population size of people aged 65 years and older. Additionally, predictors that worked for predicting a lower number of heatstrokes of hospital admission and death cases were low relative humidity, high ratio of men to women, and high mean annual taxable income. We also showed heatmaps for the number of heatstrokes of hospital admission and death cases observed and predicted by the best GAM in Supplementary Fig. 7, which was summed up across the entire period in the testing dataset. The searched space of hyperparameters and selected hyperparameters are shown in Supplementary Table 4.

## Discussion

The present study developed prediction models for the number of all heatstroke cases and heatstrokes of hospital admission and death cases per city per 12 h in Japan, which had high predictabilities enough to be implemented in public health settings. Compared with the GLMs using WBGT as the only predictor, machine learning models with multivariable predictors had higher prediction performances, showing the usefulness of not only WBGT but also other variables in predicting heatstroke cases. Additionally, the hybrid model consisting of the GAM and under-sampling XGBoost model had high prediction performance for spikes in the number of all heatstroke cases. Spikes in the number of heatstrokes of hospital admission and death cases were predicted well by the GAM. If an alert system is socially implemented based on the present prediction models, many citizens will be informed of the daily risk of heatstroke and try to prevent it by themselves, and medical staff could support self-management for high-risk citizens. Additionally, we believe that the prediction models can be useful in optimizing medical resources such as the assignment of medical staff and ambulances in emergency medicine and public health settings, which is in need of future investigations.

The present study showed the importance of multivariable predictors as well as WBGT in predicting the number of heatstroke cases. WBGT is a well-known index and traditionally used to assess heatstroke risk^8,10^. However, WBGT does not reflect heat strain adequately when the evaporation of sweat is restricted by high humidity or low air movement^7,13^. Additionally, WBGT is not precise enough to stratify the heatstroke risk in Japan as there have been many days in July and August warned by WBGT criteria proposed in the guideline for the prevention of heat disorder^10^. In the present training (i.e., 2015–2017) and testing (i.e., 2018) datasets, there were, respectively, 64.2% and 62.7% days for “Severe warning (28–31 °C), and 24.5% and 30.2% days for “threat of heat disorder (higher than 31 °C) in August. Furthermore, previous studies showed that factors other than WBGT were associated with heatstroke. The number of heatstroke patients was reported to spike at the end of the rainy season^12,14^. Heat waves, consecutive days with extremely high temperature, were associated with hospitalization due to heatstroke and heat-related mortality^15,16^. Similar variables to these factors were used in our prediction models with multivariable predictors, which allowed us to solve the WBGT limitation and predict the number of heatstroke cases more accurately compared to GLMs with WBGT only. Note that we developed the hybrid model consisting of the GAM and the under-sampling XGBoost model to predict spikes in the number of all heatstroke cases, of which prediction accuracy was much better compared to the GAM alone and the under-sampling XGBoost model alone (Figs. 1, 2). In the best GAM for the number of heatstrokes of hospital admission and death cases, under-sampling and bagging techniques were unnecessary. Its possible reasons could be as follows. Differences in the maximum total daily number of heatstrokes of hospital admission and death cases across the 16 cities between the training and the testing datasets was just 25 cases while that was 105 cases for all heatstroke cases. Thus, the best GAM without under-sampling and bagging techniques could adequately predict spikes.

For the present prediction models, we observed differences of the prediction performances between 2015, 2016, and 2017 (i.e., the training dataset), and 2018 (i.e., the testing dataset). We considered the possible reason for the differences as follows. There were more days for “threat of heat disorder (higher than 31 °C) in 2018 than in 2015, 2016, and 2017 based on WBGT (43.1% vs 6.0% in July). The hotter thermal condition increases the risk of heatstroke incidence^15,16^. In fact, the number of heatstrokes was higher in 2018 than in 2015, 2016, and 2017 (incidence rate [95% CI]: 74.4 [72.7–76.1] vs 37.5 [36.8–38.2] for all heatstroke cases, and 19.6 [18.8–20.5)] vs 11.0 [10.6–11.3] for heatstrokes of hospital admission and death cases). Note that our prediction models showed high prediction performances not only between 2015 and 2017 but also in 2018.

Previous studies developed prediction models with relatively high prediction performances for the daily number of heat-related illnesses or heatstrokes by using weather information in 3 Japanese prefectures^12^ and in 7 Chinese cities^11^. However, the previous prediction models were limited to daily-unit predictions, even though heatstroke is strongly influenced by weather conditions that differ from day to nighttime. Additionally, the two previous prediction models cannot estimate the number of patients by severity^11,12^. Furthermore, the previous models did not focus on predictabilities on days when the number of heatstroke cases spiked^11,12^, though it is the key to predict the days when the number of heatstrokes spike in public health settings. The previous model in Japan focused on the elderly who experienced heat-related illnesses at their home. We could solve these limitations by using the database based on individual-patient-unit reports of heatstroke incidence, and high-resolution weather information, and machine learning methods. Note that it was difficult to compare prediction performances between the previous and present prediction models due to the prediction unit differences (i.e., per day versus 12 h, and per prefectures versus cities) and due to differences between Japan and China (e.g., population size, weather conditions, and medical systems).

Regarding the important predictors of heatstroke in the present models, predictors that worked for predicting a higher number of heatstroke cases were high temperature, small difference in maximum temperature for a 12-h time frame minus its previous 24 h (i.e., consecutive hot days), and high downward solar radiation based on SHAP values. This was supported by previous models from Japan and China^11,12^. The present SHAP values for ratio of men to women should be carefully interpreted. High ratio of men to women worked as predicting a lower number of heatstroke cases based on SHAP values (Fig. 3 and Fig. 5), though the number of heatstroke cases in men was higher than in women in Japan^9,17^. This difference could be because the present models treated the ratio of men to women while considering other characteristics of cities. For example, Osaka city had a moderate ratio of men to women (93.8 versus 92.1 as the median of the participating 16 cities), higher population size (3,543,000 versus 356,000 as the median of the 16 cities), higher population size of people aged 65 years old and older (669,000 versus 92,000 as the median of the 16 cities), and moderate mean annual taxable income (3,256,000 JPN versus 3,478,000 JPN as the median of the 16 cities). High values of population size and that of people aged 65 and older worked for predicting a higher number of heatstroke cases, while high values of mean annual taxable income worked for predicting a lower number of heatstroke cases based on SHAP values (Figs. 3, 5). Although we tried to interpret how the present two best machine learning models utilized multivariable features as non-linear terms to predict heatstroke cases by calculating SHAP values, it was difficult to interpret the contribution of the ratio of men to women in predicting the number of heatstroke cases. Note that the present study aimed to develop the prediction models for heatstroke cases, but not aimed to clarify the association of risk factors with heatstroke cases.

The present study had several limitations. First, the present data were based on 16 Japanese cities located in the western area of Japan. Second, the predictability of future heatstroke incidences will depend on the accuracy of weather data. Due to the two limitations, the present prediction models may be required to adjust several parameters when the present prediction models are applied in different areas and meteorological data. Third, the present models did not consider patients’ characteristics including age, sex, and place of heatstroke incidence (e.g., indoor or outdoor), because we focused on the number of all heatstroke cases and heatstrokes of hospital admission and death cases regardless of the patients’ characteristics, other than the severity of heatstroke. Fourth, the present prediction models may not be generalized in other countries because the definition of heatstroke was different between Japan and other countries including the US and European countries. Thus, when the present prediction models are used in countries other than Japan, they should be updated according to the varying definitions of heatstroke.

In the present study, there were the following strengths. First, we utilized a population-based database for all heatstroke patients transposed by ambulances in16 Japanese cities corresponding to around a 10,000,000 population size. This could contribute to the representativity of the present prediction models. This also allowed us to consider the number of heatstroke per city per 12 h and the severity of heatstroke, which were not considered in previous prediction models. Second, we used state-of-the-art machine learning methods including the XGBoost algorithm with the under-sampling and bagging techniques, which allowed us to improve prediction performances, especially on days when the number of heatstrokes spiked. Third, the present prediction models can predict the number of heatstrokes only using data routinely collected (i.e., weather, calendar, and other characteristics of the cities). Thus, the prediction models can easily be applied. Fourth, the present prediction models can be possibly used for other years because those accurately predicted the number of heatstroke in 2018 (i.e., the testing dataset that was not used for developing the prediction models). Note that Japan experienced abnormally high temperatures in 2018 compared to other years^1^. Thus, we believe that the present models can work well in both typical and extremely hot summers.

In conclusion, the present study developed the prediction models for the number of all heatstroke cases and heatstrokes of hospital admission and death cases per city per 12 h in Japan; especially, the present hybrid model consisting of the GAM and the under-sampling XGBoost model was able to identify days when the number of heatstroke incidences abruptly increased. For the number of heatstrokes of hospital admission and death cases, the GAM prediction worked well. Future researches should prospectively evaluate whether the use of the present prediction models can improve clinical outcomes on a city-level scale.

## Methods

### Study design

The present study used two datasets in the following 16 cities: Osaka, Toyonaka, Mino, Ikeda, Suita, Sakai, Kobe, Ashiya, Nishinomiya, Amagasaki, Akashi, Himeji, Kyoto, Uji, Muko, and Nagaokakyo located in the Kinki region in Japan. Please see Supplementary Fig. 2 for the location of the 16 cities. One dataset was based on the population-based database for all heatstroke patients transposed by ambulances between 1 June and 30 September between 2015 and 2018, which was managed by the Fire and Disaster Management Agency under the Ministry of Internal Affairs and Communications of Japan. The other dataset was based on a database for weather information between 1 January and 31 December between 2015 and 2018, provided by the Weather Company as an IBM business (Atlanta, GA, USA). Weather data from June to September were used to develop prediction models, and other weather data were used to show time-series temperature. The details are described in the sections below.

We developed prediction models for the number of heatstroke cases using the datasets between 1 June and 30 September between 2015 and 2017 as the training dataset. The developed prediction models were applied to the dataset between 1 June and 30 September in 2018 as the testing dataset, to assess whether those models were able to be used in the future data. The present study was approved by the ethics committee of the National Cerebral and Cardiovascular Center (M30-055). Note that the requirement of written informed consent was waived because the present study analyzed anonymized data only.

### Outcome measures

The primary outcome was the number of all heatstrokes per city per 12 h (e.g., one row of data corresponds to data of one city in 12 h [6:00 am to 5:59 pm], and the next one row corresponds to data of that one city during the next 12 h [6:00 pm to 5:59 am]). For the secondary outcome, we used the number of heatstrokes of hospital admission (i.e., moderate and severe cases) and death cases per city per 12 h. The outcomes were derived from the population-based database registering reports of all heatstroke patients transposed by ambulances, which is managed by the Fire and Disaster Management Agency under the Ministry of Internal Affairs and Communications of Japan. In Japan, the characteristics of all heatstroke patients transposed by ambulances between June and September are registered in the database, which includes age, sex, time of day, place of incidence, and severity of the heatstroke (mild, moderate, severe, and death). “Mild” cases did not require hospitalization. “Severe” cases required hospitalization of 3 weeks and more. “Moderate” cases were those between mild and severe cases. A “death” case was a confirmed death at an initial examination after being transposed by an ambulance.

### Weather, calendar, and city-specific demographic information

We used the following weather information: relative humidity (%), precipitation in previous 12 h (mm), wind speed (m/s), downward solar radiation (kW/m^2^), and indices related to hot weather including ambient temperature (°C) for multivariable machine learning models and WBGT (°C) for classic statistical models^18^, and heat index (°C) as a demographic variable^19^. The weather information was based on a database that the Weather Company as an IBM business provided per various grid and time interval. We treated the weather data as follows. First, we used the weather data per 4-km grid point per hour. Note that the weather data in 2015 and downward solar radiation between 2015 and 2018 per 30-km grid point per hour were used because the Weather Company did not provide those values per 4-km grid point. Second, the weather data per 4-km or 30-km grid point per hour were averaged to those per city per hour. Third, in the averaged weather data per city per hour, we calculated mean, maximum, and minimum values within the previous 24 h. Fourth, weather data per city per hour were summarized to those per city per 12 h (6:00 am to 5:59 pm, and 6:00 pm to 5:59 am) by calculating mean, maximum, and minimum values within 12 h. Fifth, we calculated differences in values for mean, maximum, and minimum for these 12-h time frames minus their previous 24 h. Thus, the weather data unit was per city per 12 h (e.g., one row of the data corresponds to data of one city in 12 h [6:00 am to 5:59 pm], and the next one row corresponds to data of that one city during the next 12 h [6:00 pm to 5:59 am]).

In the database provided by the Weather Company, WBGT was estimated by the following estimation equation^18^ using its related weather variables per 4-km grid point or per 30-km grid point (i.e., where per 4-km grid points were not available, the nearest 30-km grid point was substituted in its place.). This equation was developed and validated, which can estimate WBGT in Japan with a 1.0 °C or less bias with 98.3% to 99.8% confidence.

$${{{{{{\rm{WBGT}}}}}}}= \, 	0.735\times {T}_{a}+0.0374\times {{{{{{\rm{RH}}}}}}}+0.00292\times {T}_{a}\times {{{{{{\rm{RH}}}}}}}+7.619\times {{{{{{\rm{SR}}}}}}}\\ \, 	-4.557\times {{{{{{{\rm{SR}}}}}}}}^{2}-0.0572\times {{{{{{\rm{WS}}}}}}}-4.064$$

*T*_a_, ambient temperature (°C); RH, relative humidity (%); SR, (kW/m^2^); WS, wind speed (m/s).

We also used the following calendar variables: times of day (i.e., daytime between 6:00 am to 5:59 pm, and nighttime between 6:00 pm to 5:59 am), months, rainy season, the difference in the number of days between the corresponding day and the last day of the rainy season of each year (days before the last day of the rainy season and the last day were coded as 0), and holidays (i.e., Saturdays, Sundays, Japanese national holidays, and Obon holidays). Obon holidays were 13–15 August in which many Japanese people take days off work. The rainy season was based on the report from the Japan Meteorological Agency, Ministry of Land, Infrastructure, Transport and Tourism.

We further used the following characteristics of the 16 cities: population size of each city at daytime for 6:00 am to 5:59 pm and at nighttime for 6:00 pm to 5:59 am, median age, the ratio of men to women, population size of people aged 65 years and older, mean annual taxable income (yen, total taxable income divided by the number of taxpayers liable for income tax), and green area proportion (%, area of parks, forests, and arable land divided by city area). These variables allowed the prediction models to account for population vulnerable to heatstrokes and several spatially varying factors, which could improve prediction models. Median age, the population size of each city, and the ratio of men to women based on the national census conducted in 2015 in Japan that was reported as of October 1^st^. Mean annual taxable income was based on the report of Municipal Taxation Status in Fiscal Year 2015 from the Ministry of Internal Affairs and Communications of Japan. Area of parks in 2010 was based on the national land survey data from the Ministry of Land, Infrastructure, Transport, and Tourism. Areas of forests, arable land, and city were based on the Statistics of Prefectures, Cities, Towns and Villages from the Ministry of Agriculture, Forestry, and Fisheries of Japan.

### Statistical analyses

Characteristics of the present dataset were summarized by median and interquartile range (IQR) for continuous variables, and n and % for categorical variables. These descriptive statistics were shown stratified by the training and testing datasets. Statistical analyses and prediction model development were performed by statistical software R (version 4.0.3, The R Foundation, Vienna)^20^ and caret package^21^ (version 6.0).

### Developing procedure for prediction models

We developed the prediction models for the number of all heatstroke cases with the following three steps. Note that the same procedures were also implemented for the number of heatstrokes of hospital admission and death cases. In the 1st step, we made the prediction models (1) by using GLM assuming Poisson distribution with WBGT as the only predictor and log of population size as the offset term in which we did not use any machine learning methods including cross-validation (CV), (2) by using GLM assuming Poisson distribution with multivariable predictors and log of population size as the offset, (3) by using GAM assuming Poisson distribution with multivariable predictors and log of population size as the offset, (4) by using random forest models with multivariable predictors, and (5) by using XGBoost with multivariable predictors. Among the above five approaches, we determined the best prediction model by the least RMSE. GLM is a general linear model in which a dependent variable is linearly related to independent variables by a link function. Additionally, GLM can specify an error distribution other than the normal distribution. GAM can model non-linear associations of independent variables with a dependent variable by using spline functions. Random forest model is a tree-based model with ensemble machine learning by fitting a number of decision trees on subsamples of the training dataset and integrating their trees. XGBoost is an optimized distributed gradient boosting decision tree library. In XGBoost, a model trains a sequence of decision trees with minimizing predictive errors of existing decision trees.

In the 2nd step, we developed city-specific prediction models by using a training dataset of the corresponding city (e.g., we used the training dataset of Osaka city when developing prediction models specific to Osaka city) and by using the same model method to the best prediction model in the 1st step (i.e., one of GLM, GAM, random forest, or XGBoost). City-specific models were compared with the best prediction model common to the 16 cities (i.e., the best prediction model in the 1^st^ step) by RMSE at each city and for a combination of all the cities. This allowed us to check whether the best prediction model common to all cities in the 1st step had serious problems related to heterogeneity of the 16 cities.

Furthermore, we proceeded to the 3rd step if the best model through 1st and 2nd steps did not adequately predict spikes in the number of heatstroke cases. We applied under-sampling and bagging techniques to the best model through the 1st and 2nd steps for learning patterns in which the number of heatstrokes spiked. The present under-sampling and bagging techniques were designed to efficiently learn a cluster of the training data in which the number of heatstroke cases was large by decreasing the other cluster of the training data in which the number of heatstroke cases was small, modeled by a method for treating class imbalance^22^. For details, please see the “*Under-sampling and bagging procedure*” section in Methods.

### Feature selection and hyperparameter optimization

For each model, we used 5-fold cross-validation (CV) to select optimal predictors (i.e., feature selection) by RFE and to search optimal hyperparameters (i.e., hyperparameter optimization) by grid-search^23^. The optimal predictors and the optimal hyperparameters were selected based on the least RMSE of each model.

RFE is a wrapper-type feature selection. RFE searches a subset of predictors by first training a model by all possible predictors, ranking all possible predictors by their feature importance, selecting the top 1 to the maximum number of all possible predictors in order of importance, and making an updated model by the selected predictors, repeated until the best subset of predictors by the least prediction error (i.e., the least RMSE in the present study) are found^23^.

As all possible predictors, we used the following weather information, calendar variables, and characteristics of each city. Weather information included precipitation in previous 12 h (mm), wind speed (m/s), ambient temperature (°C), relative humidity (%), downward solar radiation (kW/m^2^), and difference values for mean, maximum, and minimum between a 12-h time frame and its previous 24 h for ambient temperature. The calendar variables consisted of times of day (i.e., daytime between 6:00 am to 5:59 pm, and nighttime between 6:00 pm to 5:59 am), months, rainy season, difference in the number of days between the corresponding day and the last day of the rainy season of each year, and holidays. The characteristics of each city included median age, population size, the population size of people aged 65 years and older, the ratio of men to women, mean annual taxable income, and green area proportion (%, area of parks, forests, and arable land divided by city area). Note that the population size (not for the population size of people aged 65 years and older) was modeled into log of population size as the offset term in GLM and GAM.

When developing the city-specific prediction models in the 2nd step of the *“Developing procedure for prediction models”* section, we performed feature selection and hyperparameter optimization as follows. Feature selection was performed in the training dataset of all cities by RFE before developing the city-specific models. The selected features were used to develop the city-specific prediction models. Hyperparameter optimization was conducted for city-specific prediction model of each city. Note that we included weather information and calendar variables, while we did not include the characteristics of each city except the log of population size as the offset term.

### Evaluation of the developed prediction models

Primarily, overall predictive accuracies of the developed prediction models were evaluated by RMSE per city per 12 h. RMSE is the square root of mean value of squared differences between observed and predicted values (i.e., errors). Simply put, RMSE shows an average predictive error. Thus, a smaller RMSE shows better prediction performance.

Secondarily, predictive accuracies for spikes in the number of heatstroke cases were evaluated by MAPE per 24 h across all cities on days when the number of all heatstroke cases, and heatstrokes of hospital admission and death cases were large. Additionally, those were evaluated by total absolute percentage error across the entire period and all cities. MAPE and total absolute percentage error were calculated after observed and predicted values were summed up per day across all cities (for MAPE) and per the entire period across all cities (for total absolute percentage error) on days when the number of all heatstroke cases was 80th percentile (corresponding to 53.6 in 2015, 57.8 in 2016, 60.6 in 2017, and 89.8 in 2018) and above in each year, and when the number of heatstrokes of hospital admission and death cases was 80th percentile (corresponding to 15.6 in 2015, 16 in 2016, 17 in 2017, and 23 in 2018) and above in each year. MAPE is a mean value of absolute errors divided by observed values. Lower MAPE means higher predictive performance. Total absolute percentage error is the absolute proportion of differences between the sum of predictive values and the sum of observed values (as the numerator) in sum of observed values (as the denominator). Lower total absolute percentage error means higher predictive performance. Note that when predicted values were under 0, the predicted values were treated as 0 on evaluating the developed prediction models. Additionally, when predicted values were over 104 for the number of all heatstroke cases and over 48 for that of heatstrokes of hospital admission and death cases, the predicted values were 104 and 48, respectively referring to the observed number of heatstrokes in the training dataset. In the training dataset, the maximum number per city per 12 h was 52 [= 104 / 2] for all heatstroke cases, and that was 24 [= 48 / 2] for heatstroke cases of hospital admission and death cases).

We investigated what predictors were important in predicting the number of all heatstroke cases and heatstrokes of hospital admission and death cases by using SHAP values that explain the predicted value of an instance by calculating the contribution of each predictor to the predicted value^24^. For a given set of values of multiple predictors, a SHAP value shows how much one variable contributes to the difference between the actual prediction and the mean prediction in the context of its interaction with the other variables of the given set.

### Under-sampling and bagging procedure

The present under-sampling and bagging techniques aimed to make the best prediction model through the 1st and 2nd steps in the *“Developing procedure for prediction models”* section learn the patterns in which the number of heatstrokes spiked if the best model did not adequately predict spikes in the number of heatstroke cases. The present under-sampling and bagging techniques were designed to efficiently learn a cluster of the training data in which the number of heatstroke cases was large by decreasing the other cluster of the training data in which the number of heatstroke cases was small, modeled by a method for treating class imbalance^22^. The details of the present under-sampling techniques for the number of all heatstroke cases were as follows. Note that we used XGBoost algorithm for under-sampling and bagging techniques because XGBoost, a tree-based model, is more robust in multicollinearity than GLMs and GAMs by their nature. Additionally, XGBoost is robust in outlier values which were included in the present under-sampling and bagging techniques especially in weather data such as heat waves (i.e., extremely high temperature) and heavy rain (i.e., extremely high precipitation).

First, we derived a portion of the training dataset in which the number of all heatstroke cases was large defined by 90–98 percentiles and greater (in 1 percentile increments from 90 to 98, the best percentile was determined by MAPE per 24 h across all cities of the under-sampling XGBoost model described below) of the number of all heatstroke cases in the training dataset of the cities with a population size >500,000 (i.e., Osaka, Sakai, Kyoto, Kobe, and Himeji). This cluster would contain characteristics in which the number of all heatstroke cases was large in the training dataset. Additionally, we randomly derived a 10% portion of the training dataset in which the number of all heatstroke cases was 0 in the training dataset of the five cities with a population size >500,000. This cluster would contain characteristics on which the number of all heatstroke cases was small.

Second, we developed a classifier model by the XGBoost algorithm in the two portions of the training dataset derived at the first step of the under-sampling procedure. This XGBoost classifier model aimed to classify the training dataset of the five cities with a population size > 500,000 into a cluster that would contain characteristics on which the number of all heatstroke cases was large (called spike cluster), and into the other cluster that would contain characteristics on which the number of all heatstroke cases was small (called no-spike cluster). When developing this XGBoost classifier, we modeled a variable representing the spike or no-spike clusters as an outcome, and variables of weather, calendar, and characteristics of each city described in the *“Feature selection and hyperparameter optimization”* section as predictors. We used 5-fold CV to search optimal hyperparameters by grid-search based on the highest classification accuracy.

Third, by the developed XGBoost classifier model, we classified the training dataset of the five cities with a population size > 500,000 into the spike cluster or the no-spike cluster. Additionally, the training data of the other 11 cities with a population size < 500,000 were classified, a priori, into the no-spike cluster due to their small population sizes.

Fourth, we conducted under-sampling and bagging^22^. From the training data with the no-spike cluster specified in the previous step, we randomly derived data with sample size 100 or 200 for under-sampling (the best sample size was determined by MAPE per 24 h across all cities), which were conducted repeatedly 10 times for bagging. Based on the under-sampled data with the no-spike cluster and the spike cluster of the training data specified in the previous step, we developed under-sampling XGBoost prediction models for the number of all heatstroke cases using the XGBoost algorithms and variables of weather, calendar, and characteristics of each city described in the *“Feature selection and hyperparameter optimization”* section as predictors, which were conducted repeatedly 10 times for bagging. We used 5-fold CV to search optimal hyperparameters by grid-search based on the least RMSE. By averaging predicted values of 10 XGBoost models with under-sampling and bagging, we obtained predicted values of the under-sampling XGBoost prediction models.

Fifth, we developed a hybrid model by combining the best under-sampling XGBoost prediction model with the best prediction model through the 1st and 2nd steps in the *“Developing procedure for prediction models”* section. Predicted values of the best GAM were used when the sum of predicted values per 24 h across all cities of the best under-sampling XGBoost model was under 150. Mean predicted values of the best GAM and the best under-sampling XGBoost model were used when the sum of predicted values per 24 h across all cities of the best under-sampling XGBoost model was between 150 and 299.99. Predicted values of the under-sampling XGBoost model were used when the sum of the predicted values per 24 h across all cities of the best under-sampling XGBoost model was 300 and over.

Reasons for why we made the hybrid model were as follows. The best under-sampling XGBoost prediction model was optimized to predict the spikes in the number of all heatstroke cases, meaning that this model might be inadequate to predict the number of all heatstroke cases at normal times other than where the spikes occur. Additionally, the best prediction model through 1st and 2nd steps in the *“Developing procedure for prediction models”* section, the best GAM, was limited to low performance only when the number of all heatstrokes cases spiked. Specifically, the best under-sampling XGBoost model had larger RMSE for overall predictive accuracies per city per 12 h, but smaller MAPE for predictive accuracies on days when the number of all heatstroke cases spiked in the training dataset. On the other hand, the best GAM had smaller RMSE, but larger MAPE in the training dataset. Furthermore, in the training dataset, the best GAM predicted the number of all heatstroke cases well when its predicted values summed up per day across all cities were under 150, but did not predict well when 150 and over, especially, 300 and over. On the other hand, in the training dataset, the best under-sampling XGBoost model predicted the number of all heatstroke cases well when its predicted values summed up per day across all cities were 150 and over, but not well when that under 150.

### Reporting summary

Further information on research design is available in the Nature Research Reporting Summary linked to this article.

## Supplementary information

Supplementary Information

Reporting Summary

## Data Availability

The data related to heatstrokes to develop our prediction models are not publicly available in order to protect the privacy of a patient, as the granularity of the data may allow re-identification. Additionally, the data related to weather information to develop our prediction models are not publicly available because the weather data were commercial products provided by the Weather Company as an IBM business. Thus, requests for the non-profit use of those data should be sent to the corresponding author Kunihiro Nishimura (knishimu@ncvc.go.jp). The data access requests will be reviewed by our institutional review board. Once approved by the board, the data access requests will be admitted. It may take about 3–5 months. The other covariates were obtained as follows. The rainy season was based on the report from the Japan Meteorological Agency, Ministry of Land, Infrastructure, Transport and Tourism (https://www.data.jma.go.jp/fcd/yoho/baiu/kako_baiu07.html). Median age, population size of each city, and ratio of men to women based on the national census conducted in 2015 in Japan that was reported as of October 1st (https://www.stat.go.jp/data/kokusei/2015/kekka.html). Mean annual taxable income was based on the report of Municipal Taxation Status in Fiscal Year 2015 from the Ministry of Internal Affairs and Communications of Japan (https://www.soumu.go.jp/main_sosiki/jichi_zeisei/czaisei/czaisei_seido/ichiran09_15.html). Area of parks in 2010 was based on the national land survey data from the Ministry of Land, Infrastructure, Transport and Tourism (https://nlftp.mlit.go.jp/ksj/gml/datalist/KsjTmplt-P13.html). Areas of forests, arable land, and city were based on the Statistics of Prefectures, Cities, Towns and Villages from the Ministry of Agriculture, Forestry and Fisheries of Japan (http://www.machimura.maff.go.jp/machi/map/map1.html).

## Code availability

Codes used in this study to develop the prediction models are provided at https://zenodo.org/badge/latestdoi/382736888.

## References

[1] Imada Y, Watanabe M, Kawase H, Shiogama H, Arai M (2019). The July 2018 high temperature event in Japan could not have happened without human-induced global warming. SOLA.

[2] Bouchama A, Knochel JP (2002). Medical progress: heat stroke. N. Engl. J. Med..

[3] Patz JA, Frumkin H, Holloway T, Vimont DJ, Haines A (2014). Climate change: challenges and opportunities for global health. JAMA.

[4] Russo S, Sillmann J, Sterl A (2017). Humid heat waves at different warming levels. Sci. Rep..

[5] IPCC, Summary for policymakers. In: *Climate Change 2014: Impacts, Adaptation, and Vulnerability. Part A: Global and Sectoral Aspects. Contribution of Working Group II to the Fifth Assessment Report of the Intergovernmental Panel on Climate Change*. (eds. Field, C. B. et al.) pp. 1–32. (Cambridge University Press, Cambridge, United Kingdom and New York, NY, USA, 2014).

[6] Vautard R (2020). Human contribution to the record-breaking June and July 2019 heatwaves in Western Europe. Environ. Res. Lett..

[7] Budd GM (2008). Wet-bulb globe temperature (WBGT)-its history and its limitations. J. Sci. Med. Sport.

[8] Bröde P (2012). Deriving the operational procedure for the Universal Thermal Climate Index (UTCI). Int. J. Biometeorol..

[9] Kondo Y (2019). Comparison between the Bouchama and Japanese Association for Acute Medicine Heatstroke Criteria with Regard to the Diagnosis and Prediction of Mortality of Heatstroke Patients: a multicenter observational study. Int. J. Environ. Res. Public Health.

[10] Asayama, M. Guideline for the prevention of heat disorder in Japan. *Glob. Environ. Res*. **13**, 19–25 (2009).

[11] Wang, Y. et al. A random forest model to predict heatstroke occurrence for heatwave in China. **650**, 3048–3053 (2019). 10.1016/j.scitotenv.2018.09.369 30373081

[12] Kodera S (2019). Estimation of heat-related morbidity from weather data: a computational study in three prefectures of Japan over 2013–2018. Environ. Int..

[13] d’Ambrosio Alfano FR, Malchaire J, Palella BI, Riccio G (2014). WBGT index revisited after 60 years of use. Ann. Occup. Hyg..

[14] Miyake Y (2012). Heat related illness in Japan: the final report of Heatstroke STUDY 2010. Nihon Kyukyu Igakukai Zasshi.

[15] Bobb JF, Obermeyer Z, Wang Y, Dominici F (2014). Cause-specific risk of hospital admission related to extreme heat in older adults. JAMA.

[16] Xu Z, FitzGerald G, Guo Y, Jalaludin B, Tong S (2016). Impact of heatwave on mortality under different heatwave definitions: a systematic review and meta-analysis. Environ. Int..

[17] Ono M (2013). Heat stroke and the thermal environment. Jpn. Med. Assoc. J..

[18] Ono M, Tonouchi M (2014). Estimation of wet-bulb globe temperature using generally measured meteorological indices. JAPANESE J. Biometeorol..

[19] Rothfusz, L. P. The heat index equation (or more than you ever wanted to know about heat index). *National Weather Service. Southern region Technical attachment.* SR/SSD 90–23 (National Weather Service, 1990).

[20] R Core Team. *R: A Language and Environment for Statistical Computing* (R Core Team, 2020).

[21] Kuhn M (2008). Building Predictive Models in R Using the caret Package. J. of Statistical Software.

[22] Wallace, B. C., Small, K., Brodley, C. E. & Trikalinos, T. A. Class imbalance, redux. In: *Proc. IEEE International Conference on Data Mining, ICDM* 754–763 (2011). 10.1109/ICDM.2011.33

[23] Chen, Q., Meng, Z., Liu, X., Jin, Q. & Su, R. Decision variants for the automatic determination of optimal feature subset in RF-RFE. *Genes (Basel)*. **9**, 301 (2018). 10.3390/genes9060301 PMC6027449 29914084

[24] Lundberg, S. M. & Lee, S.-I. in: *Advances in Neural Information Processing Systems 30* (eds Guyon, I. et al.) 4765–4774 (Curran Associates, Inc., 2017).

