# Supplementary material for: Heatstroke predictions by machine learning, weather information, and an all-population registry for 12-hour heatstroke alerts
Source: Nat Commun. 2021 Jul 28;12:4575. doi: 10.1038/s41467-021-24823-0 (PMC8319225; doi:10.1038/s41467-021-24823-0)
Supplement: Supplementary file 1 — Supplementary Information [file 41467_2021_24823_MOESM1_ESM.pdf]

Supplementary information for "Heatstroke predictions by machine learning, weather information, and an all-population registry for 12-hour heatstroke alerts"

Contents

Supplementary Figure 1 to 7

Supplementary Table 1 to 3

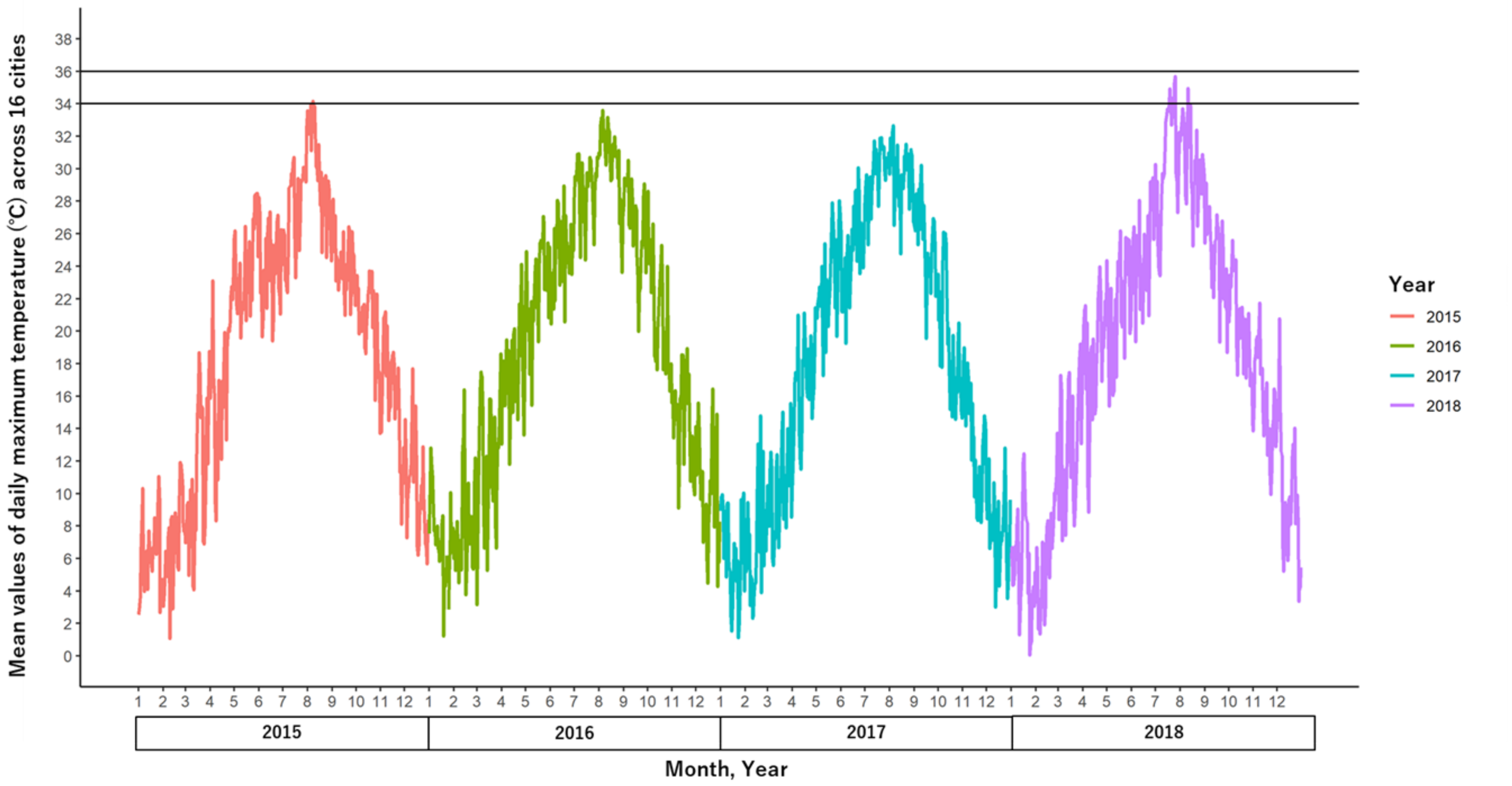

Supplementary Figure 1. Mean values of daily maximum temperature across 16 participating cities between 2015 and 2018

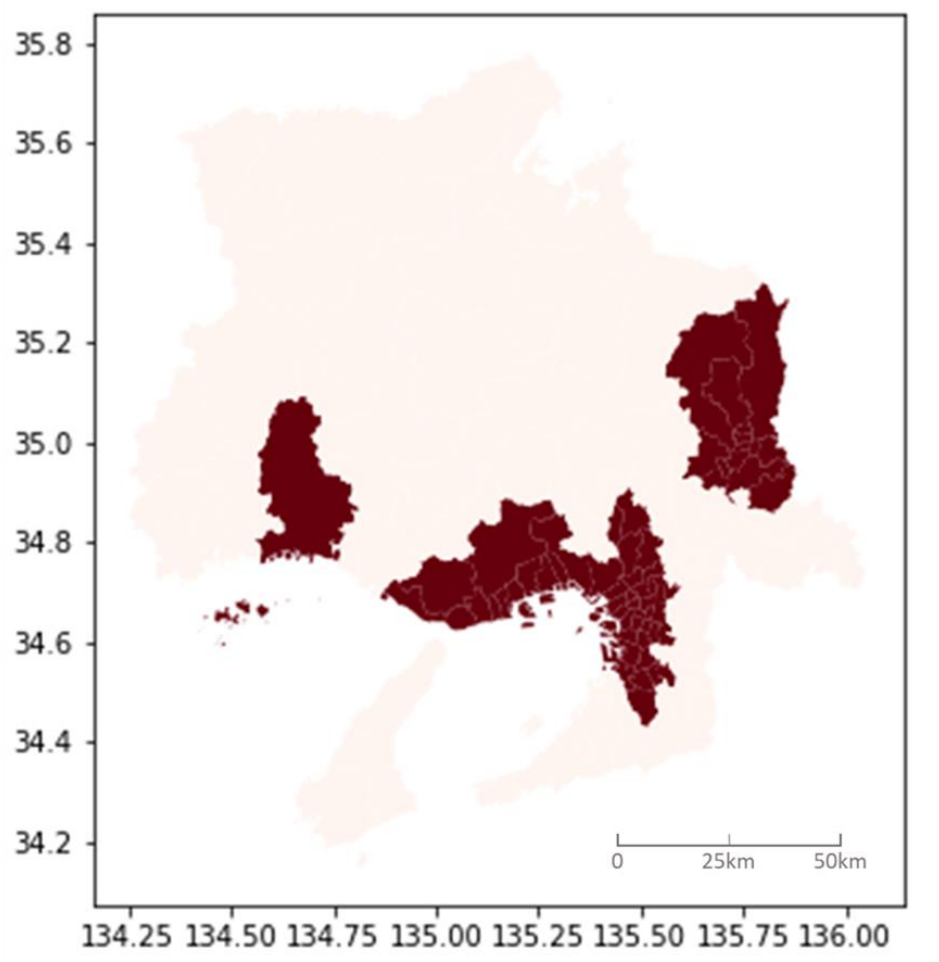

Supplementary Figure 2. Location of the participating 16 cities in Japan

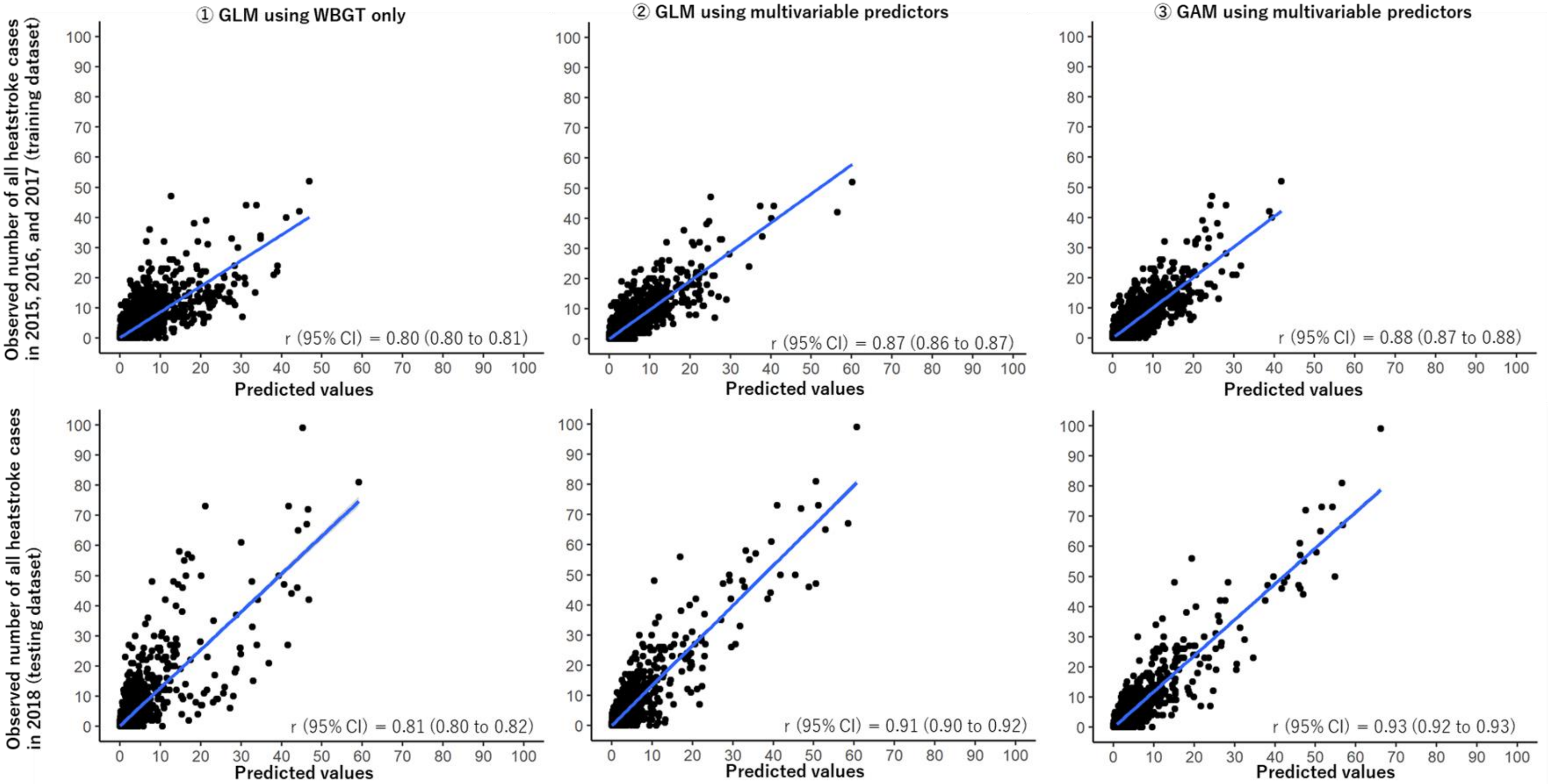

Supplementary Figure 3. Plots between observed and predicted numbers of all heatstroke cases in 2015, 2016, and 2017 (i.e., training dataset, upper row), and 2018 (i.e., testing dataset, lower row) by GLMs and GAM

Blue lines indicate regression lines between the observed and predicted values. Abbreviations: GLM, generalized linear model; WBGT, wet bulb globe temperature; GAM, generalized additive model; r, correlation coefficient; CI, confidence interval.

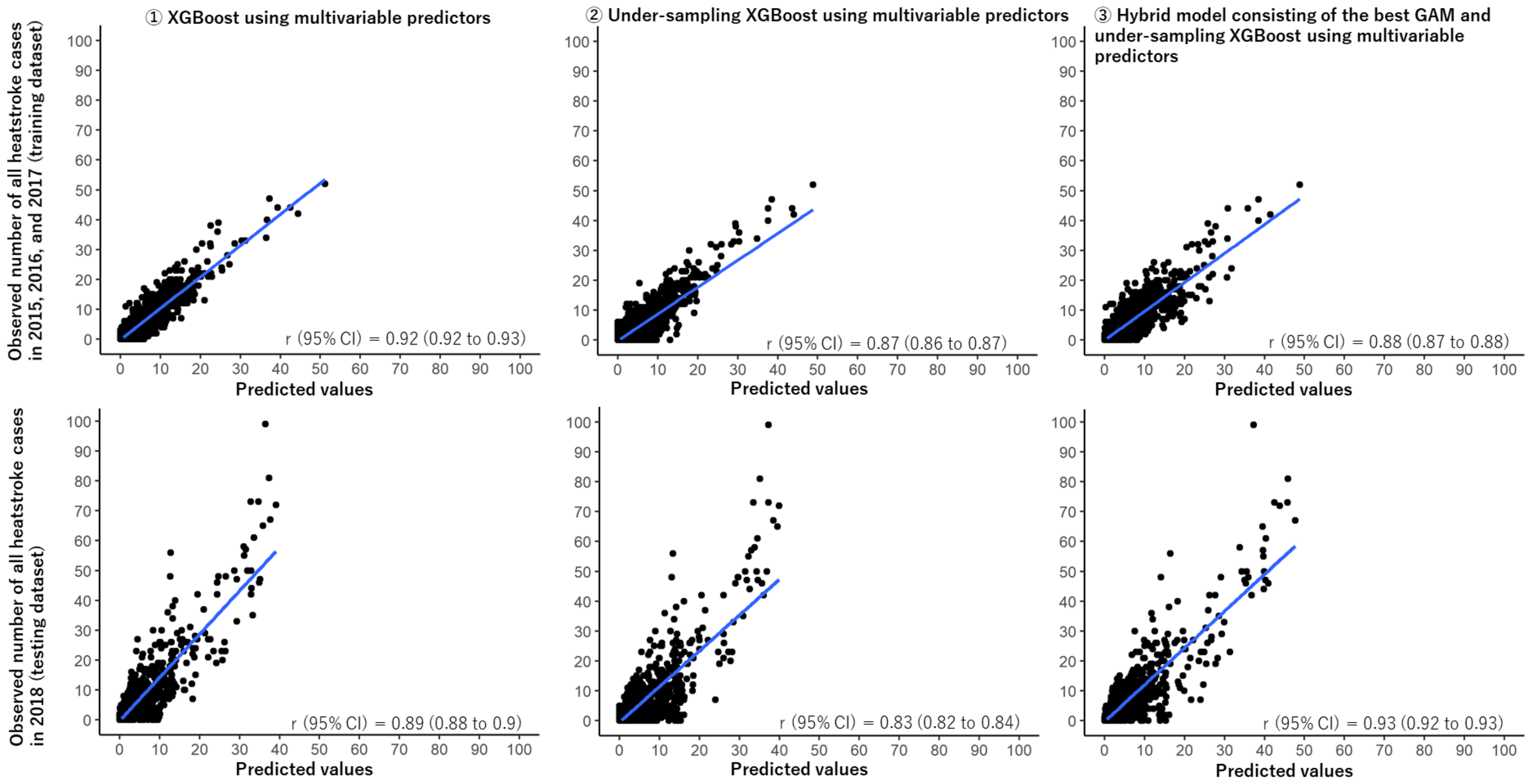

Supplementary Figure 4. Plots between observed and predicted numbers of all heatstroke cases in 2015, 2016, and 2017 (i.e., training dataset, upper row), and 2018 (i.e., testing dataset, lower row) by XGBoost models and hybrid model

Blue lines indicate regression lines between the observed and predicted values. Abbreviations: GAM, generalized additive model; XGBoost, extreme gradient boosting decision tree; r, correlation coefficient; CI, confidence interval.

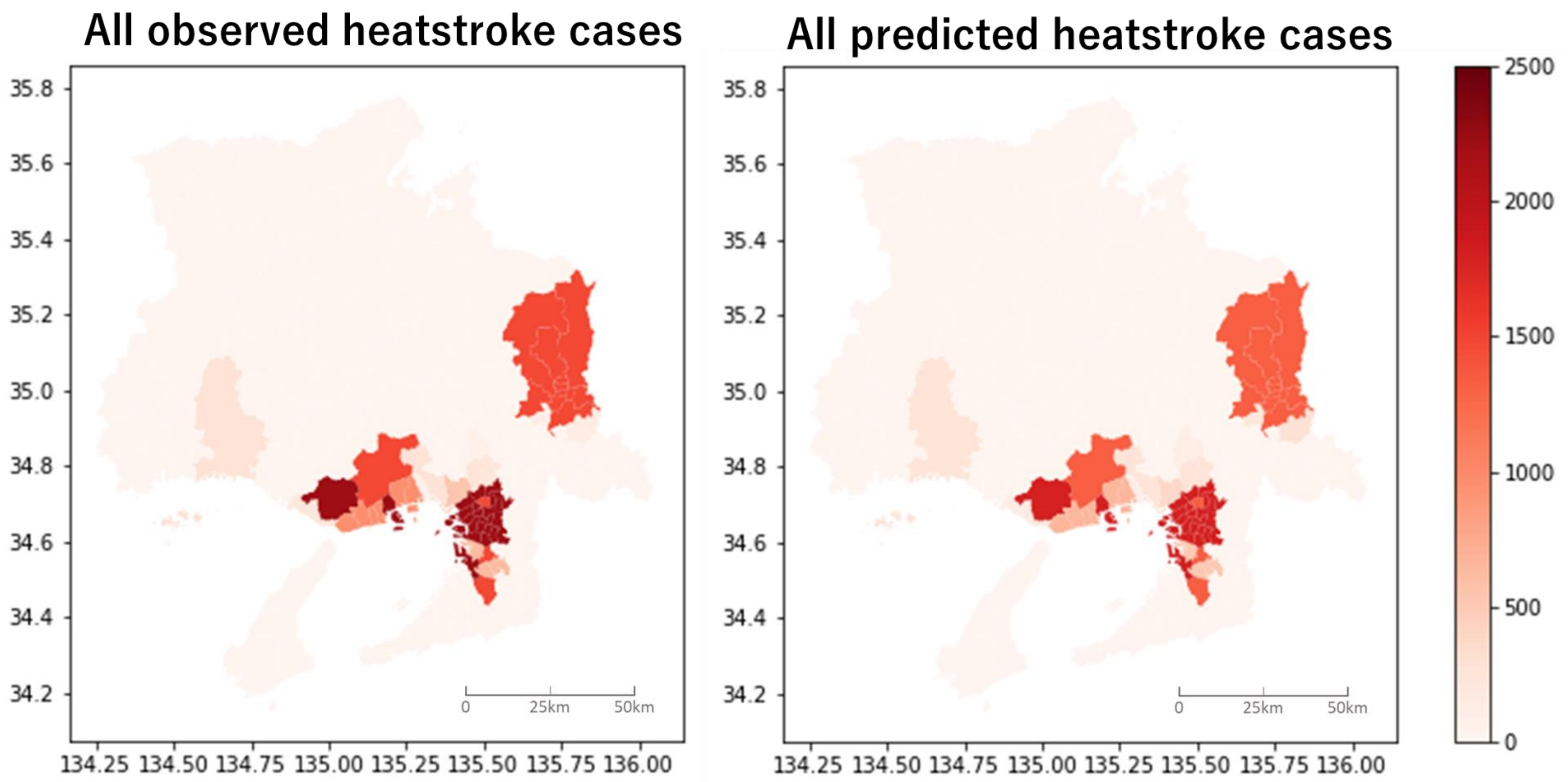

Supplementary Figure 5. Heat map for observed and predicted (by the best model) numbers of all heatstroke cases summed up across the entire period in 2018 (i.e., the testing dataset)

The low to high intensity of red color show low to high values of the number of heatstroke in the best prediction models (i.e., the hybrid model consisting of the GAM and the under-sampling XGBoost model) in a map for the 16 participating cities. The x and y-axis show latitude and longitude, respectively. Abbreviations: GAM, generalized additive model; XGBoost, extreme gradient boosting decision tree.

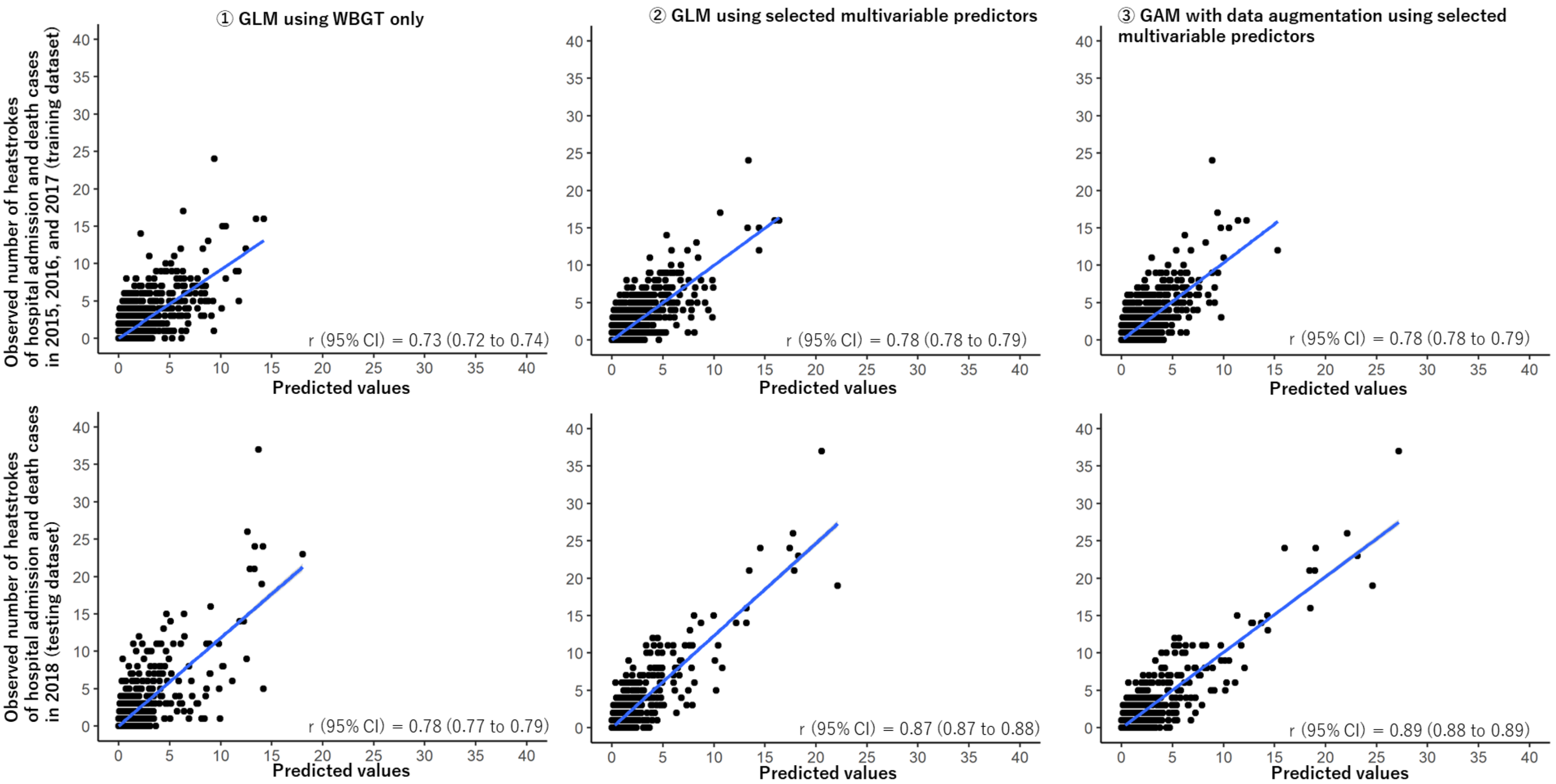

Supplementary Figure 6. Plots between observed and predicted numbers of heatstrokes of hospital admission and death cases in 2015, 2016, and 2017 (i.e., training dataset, upper row), and 2018 (i.e., testing dataset, lower row) by GLMs and GAM

Blue lines indicate regression lines between the observed and predicted values. Abbreviations: GLM, generalized linear model; WBGT, wet bulb globe temperature; GAM, generalized additive model; r, correlation coefficient; CI, confidence interval.

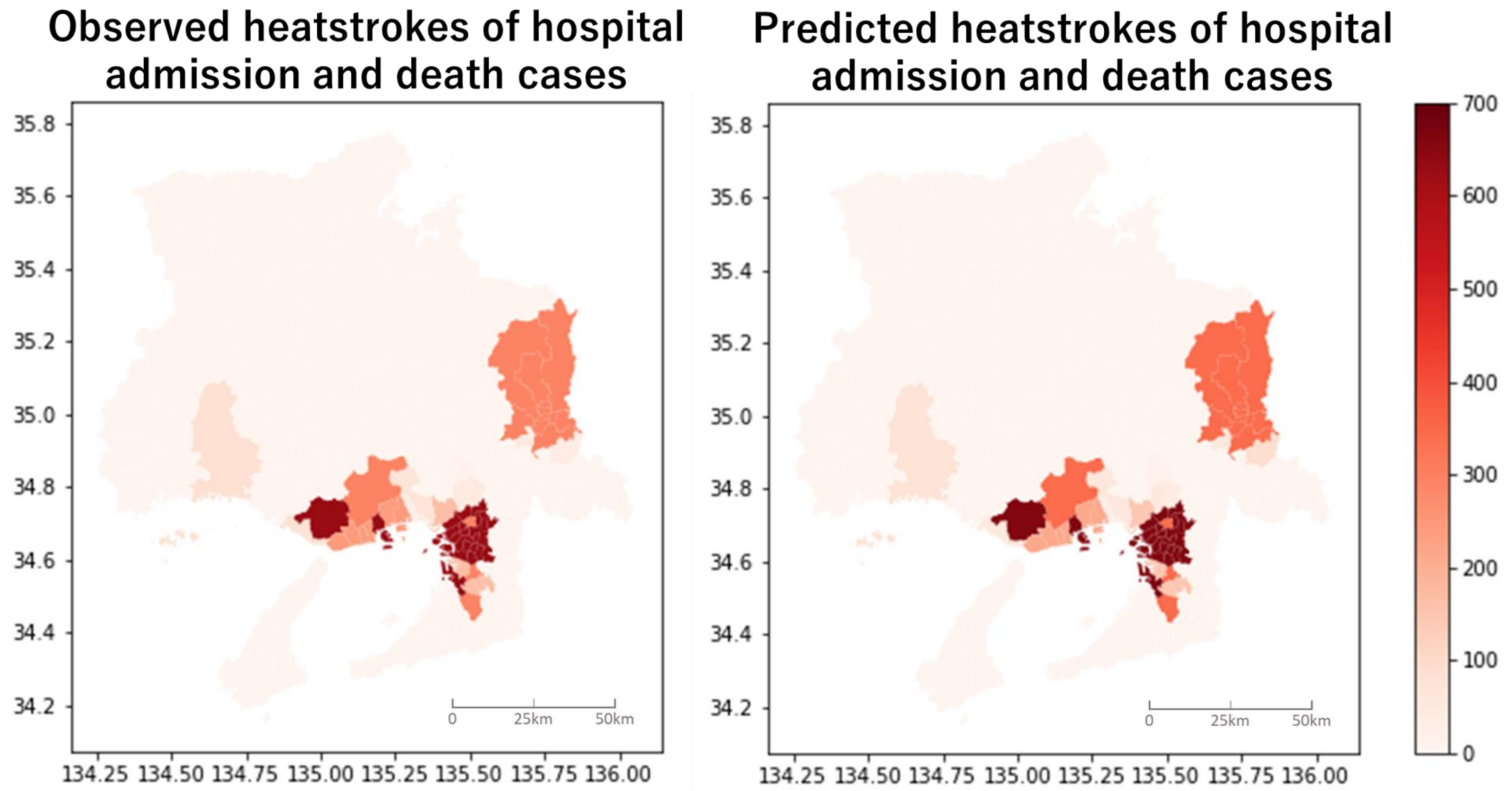

Supplementary Figure 7. Heat map for observed and predicted (by the best model) numbers of heatstrokes of hospital admission and death cases summed up across the entire period in 2018 (i.e., testing dataset)

The low to high intensity of red color show low to high values of the number of heatstrokes in the best prediction models (i.e., the GAM using multivariable predictors) in a map for the 16 participating cities. The x and y-axis show latitude and longitude, respectively. Abbreviations: GAM, generalized additive model.

Supplementary Table 1. Mean (standard deviation)[1] values of weather information per 12 hours in 16 Japanese cities between June and September from 2015 to 2018

	2015-2017 in the training dataset			2018 in the testing dataset		
	Temperature, °C	Relative humidity, %	WBGT, °C	Temperature, °C	Relative humidity, %	WBGT, °C
Kobe	24.93 (3.16)	75.7 (11.28)	23.47 (3.44)	25.36 (3.48)	75.3 (11.11)	23.76 (3.48)
Ashiya	25.12 (3.24)	73.53 (11.53)	23.38 (3.43)	25.74 (3.62)	72.34 (11.54)	23.78 (3.48)
Nishinomiya	25.32 (3.24)	72.21 (11.77)	23.43 (3.42)	25.94 (3.72)	71.13 (11.87)	23.82 (3.51)
Amagasaki	25.93 (3.3)	69.87 (12.02)	23.74 (3.4)	26.71 (3.78)	68.84 (12.02)	24.3 (3.49)
Akashi	25.23 (3.29)	75.33 (10.98)	23.7 (3.49)	25.82 (3.46)	75.74 (10.58)	24.23 (3.48)
Himeji	24.5 (3.47)	78.24 (11.1)	23.31 (3.62)	25.13 (3.82)	78.5 (11.71)	23.89 (3.72)
Kyoto	24.44 (3.63)	74.32 (12.55)	22.75 (3.6)	25.45 (4.24)	70.58 (13.31)	23.24 (3.74)
Uji	25.09 (3.71)	72.27 (12.76)	23.16 (3.6)	26.47 (4.31)	68.56 (13.19)	23.99 (3.73)
Muko	25.32 (3.66)	71.3 (12.68)	23.3 (3.57)	26.64 (4.33)	67.99 (13.23)	24.1 (3.78)
Nagaokakyo	25.18 (3.64)	72.09 (12.67)	23.25 (3.57)	26.4 (4.29)	68.64 (13.21)	23.95 (3.75)
Osaka	25.88 (3.4)	70.05 (11.91)	23.74 (3.44)	26.78 (3.89)	69.53 (12.02)	24.46 (3.55)
Toyonaka	25.78 (3.35)	69.59 (12.51)	23.56 (3.39)	26.56 (3.93)	67.86 (12.74)	24.03 (3.53)
Mino	24.9 (3.36)	72.67 (12.64)	23.07 (3.45)	25.51 (4.01)	70.26 (13.12)	23.31 (3.6)
Ikeda	24.91 (3.28)	70.73 (12.55)	22.88 (3.38)	25.47 (3.99)	69.28 (12.95)	23.17 (3.61)
Suita	25.74 (3.38)	70.13 (12.33)	23.59 (3.42)	26.67 (4)	68.11 (12.74)	24.16 (3.58)
Sakai	25.67 (3.4)	71.48 (11.91)	23.71 (3.48)	26.61 (3.87)	70.74 (11.8)	24.43 (3.55)

Abbreviations: WBGT, wet bulb globe temperature

[1] Mean (standard deviation) values were based on mean values per city per 12 hours (6:00 am to 5:59 pm, and 6:00 pm to 5:59 am).

Supplementary Table 2. City-specific RMSEs of prediction models for the number of all heatstrokes

	Training dataset			Testing dataset		
	GAM (common to all cities)	GAMs specific to each city	Hybrid model consisting of GAM and under-sampling XGBoost model (common to all cities)	GAM (common to all cities)	GAMs specific to each city	Hybrid model consisting of GAM and under-sampling XGBoost model (common to all cities)
Kobe	2.00	1.79	1.79	4.16	16.68	4.38
Ashiya	0.45	0.45	0.58	0.6	0.62	0.97
Nishinomiya	0.96	0.93	0.97	1.4	1.5	1.57
Amagasaki	1.28	1.2	1.24	2.28	1.76	2.15
Akashi	0.82	0.77	0.84	1.25	1.16	1.18
Himeji	0.84	0.8	0.8	1.59	1.54	1.57
Kyoto	2.58	2.44	2.27	3.79	4.26	4.83
Uji	0.71	0.7	0.68	1.11	0.98	1.83
Muko	0.52	0.46	0.76	0.98	0.64	2.08
Nagaokakyo	0.67	0.65	0.77	0.88	0.71	2.01
Osaka	3.18	2.91	2.88	6.44	5.97	7.87
Toyonaka	0.89	0.85	0.86	1.29	1.19	1.19
Mino	0.39	0.38	0.58	0.57	0.51	0.99
Ikeda	0.35	0.35	0.64	0.34	0.43	1.25
Suita	0.77	0.71	0.81	1.22	1.23	1.57
Sakai	1.43	1.35	1.35	2.52	9.75	2.52

Abbreviations: RMSE, root-mean-square error; GAM, generalized additive model.

Supplementary Table 3. City-specific RMSEs of prediction models for the number of heatstrokes of hospital admission and death cases

	Training dataset		Testing dataset	
	GAM (common to all cities)	GAMs specific to each city	GAM (common to all cities)	GAMs specific to each city
Kobe	0.93	0.89	1.14	1.18
Ashiya	0.31	0.29	0.35	0.39
Nishinomiya	0.43	0.42	0.58	0.61
Amagasaki	0.72	0.67	0.89	0.95
Akashi	0.45	0.45	0.65	0.62
Himeji	0.44	0.41	0.76	0.79
Kyoto	0.93	0.97	1.16	1.23
Uji	0.33	0.33	0.61	0.62
Muko	0.32	0.30	0.47	0.47
Nagaokakyo	0.37	0.35	0.51	0.48
Osaka	1.52	1.47	1.97	2.36
Toyonaka	0.41	0.41	0.46	0.54
Mino	0.14	0.13	0.21	0.23
Ikeda	0.13	0.12	0.18	0.18
Suita	0.26	0.24	0.40	0.42
Sakai	0.59	0.58	0.96	4.46

Abbreviations: RMSE, root-mean-square error; GAM, generalized additive model.

Supplementary Table 4. Space of hyperparameters of GAM, random forest, XGBoost, and under-sampling XGBoost[1]

Tables	Models	Hyperparameters	Range	Selected values
Table 2	GAM	Degree of freedom	1 to 3 (by increments of tenths)	3
Table 2	Random forest	mtry	2, and 3 to 19 (by increments of 2)	2
Table 2	XGBoost			
	Before selecting features by RFE	1st step: nrounds	10 to 90 (by increments of 10) and 100 to 1000 (by increments of 10)	300
		1st step: max tree depth	3, 5, 7, 9	3
		1st step: min child weight	1, 2, 3, 4, 5	1
		2nd step: gamma	0 to 0.4 (by increments of 0.01)	0.28
		3rd step: col sample by tree	0.6 to 1.0 (by increments of 0.1)	0.8
		3rd step: subsample	0.6 to 1.0 (by increments of 0.1)	0.9
		4th step: eta	0.01 to 0.1 (by increments of 0.01)	0.06
	After selecting features by RFE	1st step: nrounds	10 to 90 (by increments of 10) and 100 to 1000 (by increments of 10)	300
		1st step: max tree depth	3, 5, 7, 9	3
		1st step: min child weight	1, 2, 3, 4, 5	5
		2nd step: gamma	0 to 0.4 (by increments of 0.01)	0.02
		3rd step: col sample by tree	0.6 to 1.0 (by increments of 0.1)	0.6
		3rd step: subsample	0.6 to 1.0 (by increments of 0.1)	1
		4th step: eta	0.01 to 0.1 (by increments of 0.01)	0.1
Table 2	GAM specific to each city			
	Model development when selecting feature variables by RFE in all 16 cities	Degree of freedom	1 to 3 (by increments of tenths)	3

Model specific to each city using the selected features by RFE			
Akashi	Degree of freedom	1 to 3 (by increments of tenths)	3
Amagasaki	Degree of freedom	1 to 3 (by increments of tenths)	1.444
Ashiya	Degree of freedom	1 to 3 (by increments of tenths)	1.444
Himeji	Degree of freedom	1 to 3 (by increments of tenths)	2.111
Ikeda	Degree of freedom	1 to 3 (by increments of tenths)	1
Kobe	Degree of freedom	1 to 3 (by increments of tenths)	3
Kyoto	Degree of freedom	1 to 3 (by increments of tenths)	3
Mino	Degree of freedom	1 to 3 (by increments of tenths)	1.222
Muko	Degree of freedom	1 to 3 (by increments of tenths)	2.111
Nagaokakyo	Degree of freedom	1 to 3 (by increments of tenths)	2.111
Nishinomiya	Degree of freedom	1 to 3 (by increments of tenths)	1.666
Osaka	Degree of freedom	1 to 3 (by increments of tenths)	3
Sakai	Degree of freedom	1 to 3 (by increments of tenths)	1.444
Suita	Degree of freedom	1 to 3 (by increments of tenths)	1
Toyonaka	Degree of freedom	1 to 3 (by increments of tenths)	1.444
Uji	Degree of freedom	1 to 3 (by increments of tenths)	1.666
Table 3 Under-sampling XGBoost			
Definition of spike cluster	Percentiles of the number of all heatstroke cases in 5 cities with population size > 500,000	90 to 98 (1 percentile increments)	93
XGBoost, a classifier to classify the training dataset of the 5 cities into spike cluster or no-spike cluster	nrounds	50, 100, 150	50
	max tree depth	1, 2, 3	2

		min child weight	1	1
		gamma	0	0
		col sample by tree	0.6, 0.8	0.8
		subsample	0.50, 0.75, 1.00	0.5
		eta	0.3, 0.4	0.3
	Under-sampling	Sample size of a resample for under-sampling	100 or 200	200
	XGBoost with under-sampling[2]	nrounds	50, 100, 150	50
		max tree depth	1, 2, 3	3
		min child weight	1	1
		gamma	0	0
		col sample by tree	0.6, 0.8	0.8
		subsample	0.50, 0.75, 1.00	1
		eta	0.3, 0.4	0.3
Table 4	GAM	Degree of freedom	1 to 3 (by increments of tenths)	3
Table 4	Random forest	mtry	2, and 3 to 19 (by increments of 2)	3
Table 4	XGBoost			
	Before selecting features by RFE	1st step: nrounds	10 to 90 (by increments of 10) and 100 to 1000 (by increments of 10)	90
		1st step: max tree depth	3, 5, 7, 9	5
		1st step: min child weight	1, 2, 3, 4, 5	4
		2nd step: gamma	0 to 0.4 (by increments of 0.01)	0.32
		3rd step: col sample by tree	0.6 to 1.0 (by increments of 0.1)	1
		3rd step: subsample	0.6 to 1.0 (by increments of 0.1)	0.6
		4th step: eta	0.01 to 0.1 (by increments of 0.01)	0.08
	After selecting features by RFE	1st step: nrounds	10 to 90 (by increments of 10) and 100 to 1000 (by increments of 10)	80

	1st step: max tree depth	3, 5, 7, 9	5
	1st step: min child weight	1, 2, 3, 4, 5	2
	2nd step: gamma	0 to 0.4 (by increments of 0.01)	0.11
	3rd step: col sample by tree	0.6 to 1.0 (by increments of 0.1)	0.9
	3rd step: subsample	0.6 to 1.0 (by increments of 0.1)	0.8
	4th step: eta	0.01 to 0.1 (by increments of 0.01)	0.1
Table 4 GAM specific to each city			
Model development when selecting feature variables by RFE in all 16 cities	Degree of freedom	1 to 3 (by increments of tenths)	3
Model specific to each city using the selected features by RFE			
Akashi	Degree of freedom	1 to 3 (by increments of tenths)	1
Amagasaki	Degree of freedom	1 to 3 (by increments of tenths)	2.111
Ashiya	Degree of freedom	1 to 3 (by increments of tenths)	1.888
Himeji	Degree of freedom	1 to 3 (by increments of tenths)	1
Ikeda	Degree of freedom	1 to 3 (by increments of tenths)	1
Kobe	Degree of freedom	1 to 3 (by increments of tenths)	3
Kyoto	Degree of freedom	1 to 3 (by increments of tenths)	1
Mino	Degree of freedom	1 to 3 (by increments of tenths)	1.444
Muko	Degree of freedom	1 to 3 (by increments of tenths)	1
Nagaokakyo	Degree of freedom	1 to 3 (by increments of tenths)	3
Nishinomiya	Degree of freedom	1 to 3 (by increments of tenths)	1.444
Osaka	Degree of freedom	1 to 3 (by increments of tenths)	3
Sakai	Degree of freedom	1 to 3 (by increments of tenths)	1
Suita	Degree of freedom	1 to 3 (by increments of tenths)	1.888
Toyonaka	Degree of freedom	1 to 3 (by increments of tenths)	1

Uji	Degree of freedom	1 to 3 (by increments of tenths)	2.111

Abbreviations: GAM, generalized additive model; XGBoost, extreme gradient boosting decision tree; RFE, recursive feature elimination.

[1] We used the following functions in the "caret" R package: "gamSpline" for GAM, "rf" for random forest, and "xgbTree" for XGBoost. We used default values of hyperparameters for the functions, not shown in this table.

[2] We used a bagging technique with 10 resampling to develop under-sampling XGBoost. Thus, we showed selected hyperparameters of one of these 10 under-sampling XGBoost models.
