# Supplementary material for: Heatstroke predictions by machine learning, weather information, and an all-population registry for 12-hour heatstroke alerts
Source: Nat Commun. 2021 Jul 28;12:4575. doi: 10.1038/s41467-021-24823-0 (PMC8319225; doi:10.1038/s41467-021-24823-0)
Supplement: Supplementary file 2 — Reporting Summary [file 41467_2021_24823_MOESM2_ESM.pdf]

nature research

Corresponding author(s): Kunihiro Nishimura

Last updated by author(s): Jul 4, 2021

Reporting Summary

Nature Research wishes to improve the reproducibility of the work that we publish. This form provides structure for consistency and transparency in reporting. For further information on Nature Research policies, see our Editorial Policies and the Editorial Policy Checklist.

Statistics

For all statistical analyses, confirm that the following items are present in the figure legend, table legend, main text, or Methods section.

n/a	Confirmed	
☐	☒	The exact sample size (n) for each experimental group/condition, given as a discrete number and unit of measurement
☐	☒	A statement on whether measurements were taken from distinct samples or whether the same sample was measured repeatedly
☒	☐	The statistical test(s) used AND whether they are one- or two-sided<br>*Only common tests should be described solely by name; describe more complex techniques in the Methods section.*
☐	☒	A description of all covariates tested
☒	☐	A description of any assumptions or corrections, such as tests of normality and adjustment for multiple comparisons
☐	☒	A full description of the statistical parameters including central tendency (e.g. means) or other basic estimates (e.g. regression coefficient) AND variation (e.g. standard deviation) or associated estimates of uncertainty (e.g. confidence intervals)
☐	☒	For null hypothesis testing, the test statistic (e.g. F, t, r) with confidence intervals, effect sizes, degrees of freedom and P value noted<br>*Give P values as exact values whenever suitable.*
☒	☐	For Bayesian analysis, information on the choice of priors and Markov chain Monte Carlo settings
☒	☐	For hierarchical and complex designs, identification of the appropriate level for tests and full reporting of outcomes
☐	☒	Estimates of effect sizes (e.g. Cohen's d, Pearson's r), indicating how they were calculated

Our web collection on statistics for biologists contains articles on many of the points above.

Software and code

Policy information about availability of computer code

Data collection	To prepare the weather dataset used in the present study, the Weather Company as an IBM business used Python (version 3.6.8) to derive weather data from API of the Weather Company.
Data analysis	Statistical analyses and prediction model development were performed by statistical software R (version 4.0.3, The R Foundation, Vienna), caret package (version 6.0), and the following functions of the caret package: “gamSpline” for GAM, “rf” for random forest, and “xgbTree” for XGBoost.

For manuscripts utilizing custom algorithms or software that are central to the research but not yet described in published literature, software must be made available to editors and reviewers. We strongly encourage code deposition in a community repository (e.g. GitHub). See the Nature Research guidelines for submitting code & software for further information.

Data

Policy information about availability of data

All manuscripts must include a data availability statement. This statement should provide the following information, where applicable:

- Accession codes, unique identifiers, or web links for publicly available datasets
- A list of figures that have associated raw data
- A description of any restrictions on data availability

The data related to heatstrokes to develop our prediction models are not publicly available in order to protect the privacy of a patient. Additionally, the data related to weather information to develop our prediction models are not publicly available because the weather data were commercial products provided by the Weather Company as an IBM business. Thus, requests for the non-profit use of those data should be sent to corresponding author Kunihiro Nishimura (knishimu@ncvc.go.jp). The data access requests will be reviewed by our institutional review board. Once approved by the board, the data access requests will be admitted. It may take about 3 to 5 months. The other covariates were obtained as follows. The rainy season was based on the report from the Japan Meteorological

Agency, Ministry of Land, Infrastructure, Transport and Tourism (https://www.data.jma.go.jp/fcd/yoho/baiu/kako_baiu07.html). Median age, population size of each city, and ratio of men to women based on the national census conducted in 2015 in Japan that was reported as of October 1st (https://www.stat.go.jp/data/kokusei/2015/kekka.html). Mean annual taxable income was based on the report of Municipal Taxation Status in Fiscal Year 2015 from the Ministry of Internal Affairs and Communications of Japan (https://www.soumu.go.jp/main_sosiki/jichi_zeisei/czaisei/czaisei_seido/ichiran09_15.html). Area of parks in 2010 was based on the national land survey data from the Ministry of Land, Infrastructure, Transport and Tourism (https://nlftp.mlit.go.jp/ksj/gml/datalist/KsjTmplt-P13.html). Areas of forests, arable land, and city were based on the Statistics of Prefectures, Cities, Towns and Villages from the Ministry of Agriculture, Forestry and Fisheries of Japan (http://www.machimura.maff.go.jp/machi/map/map1.html). Codes used in this study to develop the prediction models is provided at https://zenodo.org/badge/latestdoi/382736888.

Field-specific reporting

Please select the one below that is the best fit for your research. If you are not sure, read the appropriate sections before making your selection.

☒ Life sciences ☐ Behavioural & social sciences ☐ Ecological, evolutionary & environmental sciences

For a reference copy of the document with all sections, see nature.com/documents/nr-reporting-summary-flat.pdf

Life sciences study design

All studies must disclose on these points even when the disclosure is negative.

Sample size	The present study aimed to develop prediction models for the number of heatstrokes by using datasets between June 1st and September 30th for heatstroke data, between January 1st and December 31th, and between 2015 and 2018 in the following 16 cities corresponding to around a 10,000,000 population size: Osaka, Toyonaka, Mino, Ikeda, Suita, Sakai, Kobe, Ashiya, Nishinomiya, Amagasaki, Akashi, Himeji, Kyoto, Uji, Muko, and Nagaokakyo located in the Kinki region in Japan.<br><br>We used all data between June and September in 2015, 2016, 2017, 2018 in the 16 cities. The sample size was 15616 rows (i.e., 1 row represented data per city per 12 hours), the largest possible sample size.
Data exclusions	No data exclusions
Replication	Predictabilities of the prediction models (e.g., RMSE, MAPE, and etc) were independently assessed twice. We confirm that all attempts at replication were successful.
Randomization	This study design was observational study. Therefore, we did not perform randomization. Additionally, we utilized many predictors to develop prediction models
Blinding	Blinding was not relevant to this study. The present study used two databases recording heatstroke incident and weather information. The heatstroke database was managed by the Fire and Disaster Management Agency under the Ministry of Internal Affairs and Communications of Japan. The weather database was provided by the Weather Company as an IBM business. Thus, all personal information had been deleted before we started to analyze the datasets. Additionally, we developed our prediction models based on the two databases between 2015 and 2017. And then, the developed prediction models were applied to the databases in 2018.

Reporting for specific materials, systems and methods

We require information from authors about some types of materials, experimental systems and methods used in many studies. Here, indicate whether each material, system or method listed is relevant to your study. If you are not sure if a list item applies to your research, read the appropriate section before selecting a response.

Materials & experimental systems

n/a	Involved in the study	
☒	☐	Antibodies
☒	☐	Eukaryotic cell lines
☒	☐	Palaeontology and archaeology
☒	☐	Animals and other organisms
☐	☒	Human research participants
☒	☐	Clinical data
☒	☐	Dual use research of concern

Methods

n/a	Involved in the study	
☒	☐	ChIP-seq
☒	☐	Flow cytometry
☒	☐	MRI-based neuroimaging

Human research participants

Policy information about studies involving human research participants

Population characteristics	The present study used a dataset in the following 16 cities: Osaka, Toyonaka, Mino, Ikeda, Suita, Sakai, Kobe, Ashiya, Nishinomiya, Amagasaki, Akashi, Himeji, Kyoto, Uji, Muko, and Nagaokakyo located in the Kinki region in Japan. This dataset was based on the population-based database for all heatstroke patients transposed by ambulances between June 1st and September 30th between 2015 and 2018, which was managed by the Fire and Disaster Management Agency under the Ministry of Internal Affairs and Communications of Japan. Thus, all heatstroke patients transposed by ambulances between June and September in 2015 to 2018 in the 16 cities were included regardless of age and sex.
Recruitment	The present study used two datasets in the following 16 cities: Osaka, Toyonaka, Mino, Ikeda, Suita, Sakai, Kobe, Ashiya, Nishinomiya, Amagasaki, Akashi, Himeji, Kyoto, Uji, Muko, and Nagaokakyo located in the Kinki region in Japan.<br><br>One dataset was based on the population-based database for all heatstroke patients transposed by ambulances between June 1st and September 30th between 2015 and 2018, which was managed by the Fire and Disaster Management Agency under the Ministry of Internal Affairs and Communications of Japan. The datasets of the 16 cities were provided by the cities.<br><br>The other dataset was based on a database for weather information between January 1st and December 31th between 2015 and 2018, provided by the Weather Company as an IBM business (Atlanta, GA, USA).
Ethics oversight	The present study was approved by the ethics committee of the National Cerebral and Cardiovascular Center (M30-055). Note that the requirement of written informed consent was waived because the present study analyzed anonymized data only.

Note that full information on the approval of the study protocol must also be provided in the manuscript.
